# Prediction of the performance of industrial gas turbine based on different environmental conditions

**DOI:** 10.1371/journal.pone.0358137

**Published:** 2026-09-21

**Authors:** Fizza Ghulam Nabi, Muhammad Usman, Muhammad Ehtisham, Kashif Ishfaq, Uwamahoro Raphael, Usman Aziz, Hafiz Muhammad Umar

**Affiliations:** 1 Institute of Industrial Engineering and Management, University of the Punjab, Lahore, Pakistan; 2 Department of Mechanical Engineering, University of Engineering and Technology, Lahore, Pakistan; 3 University of Engineering and Technology Lahore, Manufacturing Engineering Department UET, Lahore, Pakistan; 4 University of Rwanda, College of Science and Technology, Regional Centre of Excellence in Biomedical Engineering and e-Health, Kigali, Rwanda; Dr Shakuntala Misra National Rehabilitation University, INDIA

## Abstract

This study examines the effect of ambient environmental conditions on the performance and efficiency of General Electric GE-9171E gas turbines used for electricity generation at the Nandipur power plant. The research emphasizes the importance of improving gas turbine efficiency to reduce fuel consumption, operational costs, and greenhouse gas emissions, thereby promoting environmentally sustainable power generation in Pakistan. The study specifically evaluates the influence of ambient temperature, humidity, and dew point on turbine output and operational efficiency. Real-time operational data were collected from three gas turbines operating at full load over a one-year period. Both conventional statistical methods and artificial intelligence (AI)-based approaches were employed for performance evaluation and prediction. Conventional techniques included descriptive statistics, correlation analysis, and regression modeling, while AI-based methods incorporated support vector machine (SVM) and artificial neural network (ANN) models. The findings indicate that ambient temperature has a significant negative impact on turbine performance, showing a strong correlation with output power and efficiency (r = −0.959, R^2^ = 92.1%). Humidity and dew point also affected turbine behavior, although their relationships with performance were comparatively less pronounced. A regression-based predictive model was developed and validated using operational plant data, achieving high prediction accuracy with a minimum error of −0.01088 for Gas Turbine-I. Comparative analysis demonstrated that ANN-based models outperformed conventional techniques in prediction capability. Overall, the study provides a reliable framework for predicting gas turbine performance and improving operational efficiency under varying climatic conditions in Pakistan.

## Introduction

A gas turbine is a rotating combustion engine which uses the chemical energy contained within a fuel, and through a continuous combusted cycle of compression, combustion and expansion, converts this to mechanical and electrical energy. Gas turbines have been increasingly exploited for power generation use due to a number of laudable characteristics such as high power density, low weight, fast engine start-up and fuel versatility [1]. The concept of land based gas turbines can be traced to the very beginning in the town of Neuchtel in Switzerland in 1939, where the world’s first gas turbine for industrial use was brought online, and because of this a world altering change in the way of chemical to mechanical energy conversion [2]. Still since then gas turbines have been implemented for commercial use in an array of industries like oil and gas manufacturing heavy transport vehicles, rail infrastructure and marine propulsion, in part due to the similarity in design and through the synergism of their efficiencies with those of steam turbines as well as incorporating Really they require much shorter installation periods and exhibit relatively lower operational costs, faster start-up and higher levels of efficiency achievable than in combined cycle arrangements [3].

Although developments in the design, configuration, and material properties of gas turbines have advanced really over the last 50 years, perhaps the most significant has been the recovery of heat, allowing the turbines to operate on a closed cycle. This involves diverting the hot exhaust gases into a heat recovery steam generator to drive a steam turbine and because of this boost the overall plant efficiency to greater than 60% [4]. Businesses have been able to increase the size of turbines, like the A320’s CFM56 engines, which have found ways of increasing thrust and power generation by improving the airflow and optimizing the strength of materials without changing the original platform design [5]. Studies on the thermodynamic scaling of turbines have also shown that the power and flow increase quadratically with scale whereas the cycle efficiency remains essentially the same [6].

Fuel flexibility has emerged as a major focus of innovation in gas turbine technology. Although natural gas is still the main fuel choice because of its cleaner combustion properties, other fuels like heavy fuel oil diesel hydrogen, and biofuels bring benefits based on different regional and economic factors [1]. Researchers are in particular interested in hydrogen combustion for its role in low-emission, carbon-free energy cycles but issues such as flame speed and combustion stability are still being researched [7]. At the same time, materials science has given a big lift to turbine component durability against heat: moving from regular metallic alloys to nickel-based superalloys and ceramic matrix composites, plus the application of thermal barrier coatings, has made it possible for turbine blades to operate at higher temperatures without suffering damage [8]. And, additive manufacturing has opened the door to very detailed internal cooling channel shapes that enhance cooling efficiency beyond previous limits while ensuring mechanical precision [9].

Nevertheless, such technological breakthroughs, the performance of gas turbines continues to be heavily influenced by changes in the external environment. The international organization for standardization has fixed operational parameters for performance rating, e.g., outdoor temperature at 15°C, air humidity at 60%, and pressure at 1 atmosphere. Still, in tropical and subtropical regions like Pakistan, operating conditions often vary a lot and continuously from these standards, and reductions in power output of around 20% have been recorded at the most extreme summer conditions [10,11]. By far, the most important environmental factor from a thermodynamics standpoint influencing gas turbine power output is ambient temperature. Gas turbines basically work on the principle of mass flow: when the outside temperature goes up, the air density goes down per the ideal gas law, so the mass flow rate of air that gets into the compressor is also lower. This decrease in air flow results in a lower pressure ratio being generated, less energy being produced in the combustion chamber, and eventually a decrease in the power output. Studies on the matter have shown that the efficiency of a gas turbine drops by around 0. 1% for every degree Celsius increase in ambient temperature above the ISO baseline level, and power output losses as high as 19% have been demonstrated when the outdoor temperature rises from 14°C to 40°C [12,13].

Humidity has a minor but noticeable effect on the performance of gas turbines. When there is more water in the air, the amount of oxygen available for combustion per volume of air decreases and the specific heat capacity of the work fluid changes a little, which in turn influences combustion temperature and flame stability. One study found that compressor specific power is reduced by 6.18% when relative humidity changes from 10% to 90% at 35°C [14]. Also, a more gradual increase in relative humidity from 15% to 95% resulted in power output reduction by only 0. 15% to 0. 77%, according to the ambient temperature [16]. This shows that although humidity is a relevant predictor statistically, its thermodynamic effect is quite a bit less than that of ambient temperature. Dew point temperature is the temperature at which air is saturated with water vapor. It gives a direct measure of the absolute moisture loading and is independent of ambient temperature. If ambient temperature comes close to the dew point, condensation may take place at the compressor inlet, thereby changing effective mass flow and blade aerodynamics. That is why dew point is a good thermodynamic indicator of the quality of inlet air.

Pakistan’s climate is such that dew point and its role in gas turbine performance will be very important there. In fact, dew point is crucial in an area like Pakistan where the climate is both humid and variable [15]. Since high ambient temperature and humidity have a negative impact on performance, researchers have looked at ways of cooling the inlet air such as fogging, evaporative cooling, and chiller-based turbine inlet air cooling systems. These systems have been demonstrated mostly to restore air density and because of this increase the power output by 10% to 30%, the exact figure according to the specific technology and local climatic conditions [16,17]. Over the last decade, the use of data-driven and machine learning techniques to model the performance of gas turbines has increased a lot. Initially, regression and correlation methods were used to set up the field and show the potential. For example, by using linear regression on operational data of an MHI gas turbine, the power drop resulting from a one-degree rise in ambient temperature proved to be 1. 36 MW. This figure was later used as a basis for a degree-day approach to estimate the maximum power production capability theoretically [18]. Besides, Tufekci proved that multiple machine learning regression methods given ambient temperature, atmospheric pressure, relative humidity, and exhaust vacuum as inputs and six years of combined cycle power plant data could amazingly beat simple statistical regression models in predicting full load electric power output [19].

One of the contributions of advanced mathematical setups, to the theoretical understanding of heat and mass transport things, has been through the creation of fractional derivative models that enable us to comprehend thermally stratified and transient flow behaviors in combustion and heat exchange situations. For example, the studies of unsteady magnetohydrodynamic flow of second-grade fluids that incorporate fractional derivative formulations are able to shed light on these aspects of the flow behavior [12,20]. Besides, bio-convective fractional flow models are another case of extending the range of generalized governing equations to describe the complex thermal and mass transfer occurrences in vertical channel geometries [21]. The use of constant proportional caputo fractional models for carbon nanotube-based heat transport systems has revealed that nano- enhanced fluids having fractional-order constitutive relations perform heat transfer more efficiently, which could be considered for advanced cooling applications in thermal machinery [22]. The study of fractionalized Brinkman flow including diffusion effects have added to the supply of mathematical methods for describing viscous and thermally driven flows in industrial systems [23].

Besides forecasting, optimization methods have also been implemented to improve the performance of gas turbines in different working conditions. Soft computing techniques like genetic algorithms, particle swarm optimization, fuzzy logic, and adaptive neuro-fuzzy inference systems have been utilized for optimizing turbine design, blade shapes, and operational parameters. A thorough review of soft computing methods for optimizing the thermal performance of gas turbines showed that particle swarm optimization outperformed other methods like genetic algorithms and simulated annealing on faster convergence and better solution quality [24]. When combined with particle swarm optimization, physics-informed neural networks have reached R^2^ 94. 03% simultaneously maximizing turbine power and minimizing specific fuel consumption [25]. Also, digital twin technologies that blend physical system simulations with instantaneous data-driven predictions have been suggested for diagnosing gas turbine conditions, providing early warnings of performance faults, and detecting anomalies. These represent the leading edge of intelligent gas turbine control and a logical progression from the prediction models of the present research [26–28]. The theoretical basis for using ANN in thermal-fluid prediction has been recently supported and enhanced by studies that explore fractional-order operator modeling of heat and mass transport. Research applying fractional operators alongside ANN for nanofluid flow prediction has shown that these hybrid methods can precisely represent nonlinear temperature and concentration distributions, with errors in predictions being less than the threshold levels even for highly nonlinear thermal systems [29].

Data-driven and physics-informed modeling approaches have been broadly applied across diverse engineering domains, including power electronics and inverter control systems [30], plasma discharge simulation [31], advanced materials characterization [32], thermal contact conductance prediction [33], geothermal energy systems [34], and wellbore heat transfer modeling [35], collectively demonstrating the versatility and growing adoption of computational and intelligent frameworks in complex engineering applications.

Work on unsteady magnetohydrodynamic flow of second-grade fluids using two different fractional derivative expressions has clarified thermally stratified flow characteristics that are pertinent to combustion atmospheres [36,37]. Bio-convective flow studies blending Fick’s and Fourier’s laws within fractionalized transport models have also demonstrated how generalized governing equations can be used to describe transient thermal and mass transfer events [38]. Besides, constant proportional Caputo fractional approach to carbon nanotube-based heat transport systems suggested that nano-fluid with fractional-order constitutive relations might give enhanced heat transfer performance which is something important for turbine blade cooling [39]. Further research works on fractionalized Brinkman flow with diffusion effects, fractional magnetohydrodynamic Casson fluid flow under thermal radiation and buoyancy, and chemical reaction effects on magnetohydrodynamic. Casson flow using Caputo fractional derivatives strengthen the mathematical depth of nonlinear thermal-fluid prediction setups that are foundational for contemporary data-driven methods in industrial heat transfer including those applied to gas turbine thermodynamic cycles [37,40–42].

Similar statistical and machine learning approaches to the ones described here have been successfully applied in other engineering and biomedical domains, indicating the relative maturity of these methodological approaches and their applicability. Statistical and machine learning analysis was used to classify levels of multi-level disease severity based on acoustic signal data to achieve high correlation with observed severity levels [43], integrated power feature extraction and a classifier was used to extract severity levels from physiological sound recordings [44], software applications implementing that classification schemes were used to identify severity levels of a condition in a clinical setting [45], and multi-scale deep learning segmentation schemes were used to differentiate severity levels for complex image-based diagnostic tasks [46]. These related studies provide methodological precedent for the use of statistical correlation and regression, coupled with machine learning classifiers, for severity level prediction where originally established for a different application domain. Despite this significant body of literature on gas turbine power prediction, the body of knowledge present continues to demonstrate a few key gaps in the literature. So far, most known published prediction studies have been based on test data obtained near ISO conditions from temperate climates and used only ambient air dry-bulb temperature and relative humidity as predictor variables (without consideration of dew point as an independent predictor).

Also, the existing published works in the literature use only a single turbine machine in their prediction models and none so far have reported a comparative analysis of the prediction performance of an ANN versus a SVM for the same gas turbines power output prediction problem for data collected in South Asia. This research seeks to fill this gap in the literature by examining the effects of all three ambient variables namely ambient temperature, dew point and relative humidity on the active power produced by three gas turbines 1, 2 and 3 (denoted as GT I, GT II and GT III), at Nandipur power plant, Pakistan. To this end, correlation and regression analysis are utilized to statistically determine the effects (through individual relationship) of each of the three ambient air variables on active power, and ANN and SVM power prediction schemas are developed and statistically evaluated against each other. A power estimation calculator is also constructed which can be used to estimate turbine power for operational planning and scheduling purposes. The specific novelty and unique contributions of this work are like this: (i) incorporates effect of dew point as an influential predictor variable, operating in a real, non-ISO operating environment of a South Asian station; (ii) models active power from three turbines simultaneously, because of this allowing comparative analysis of power prediction robustness between turbines which have different operational histories; and (iii) Implementation of machine learning techniques SVM and ANN to predict the performance of gas turbine under different environmental conditions. Finally, the accuracy of the regression model will be validated. The data obtained is feasible for planning and scheduling the upcoming operational details in future.

## Methodology

In this study real-time data has been collected from one the largest power generating source of Pakistan. Genco holding company (GENCOS) is one of the largest thermal power generation companies in Pakistan. GENCOS is further divided into 4 sub companies, GENCO I, GENCO II, GENCO III and GENCO IV. In this research paper our focus is on GENCO-III, also named as Northern power generation company limited (NPGCL) in Pakistan. NPGCL has total installed capacity of 2059.65MW which consists of Shadra, Nandipur and Muzaffargarh plants. Nandipur power plant Gujranwala 425–525MW is located on the bank of Upper Chenab Canal (UCC) and this project 12 KM apart from Gujranwala City, Pakistan. Nandipur Power Plant consists of three General Electric (GE) Frame 9E Gas turbines (GE-9171E) and one Dongfang electric steam turbine.

In this study data has been collected from Nandipu Power plant by one of the authors Muhammad Ehtisham, also served in plant as an Instrumentation and Control Engineering. Permission for data collection and it use for research has been obtained from the plant Manager Nandipur Power Plant Gujranwala, Genco 3. The raw data used in this study is available on the Zenodo website: https://zenodo.org/records/22237139.

**Fig 1:**
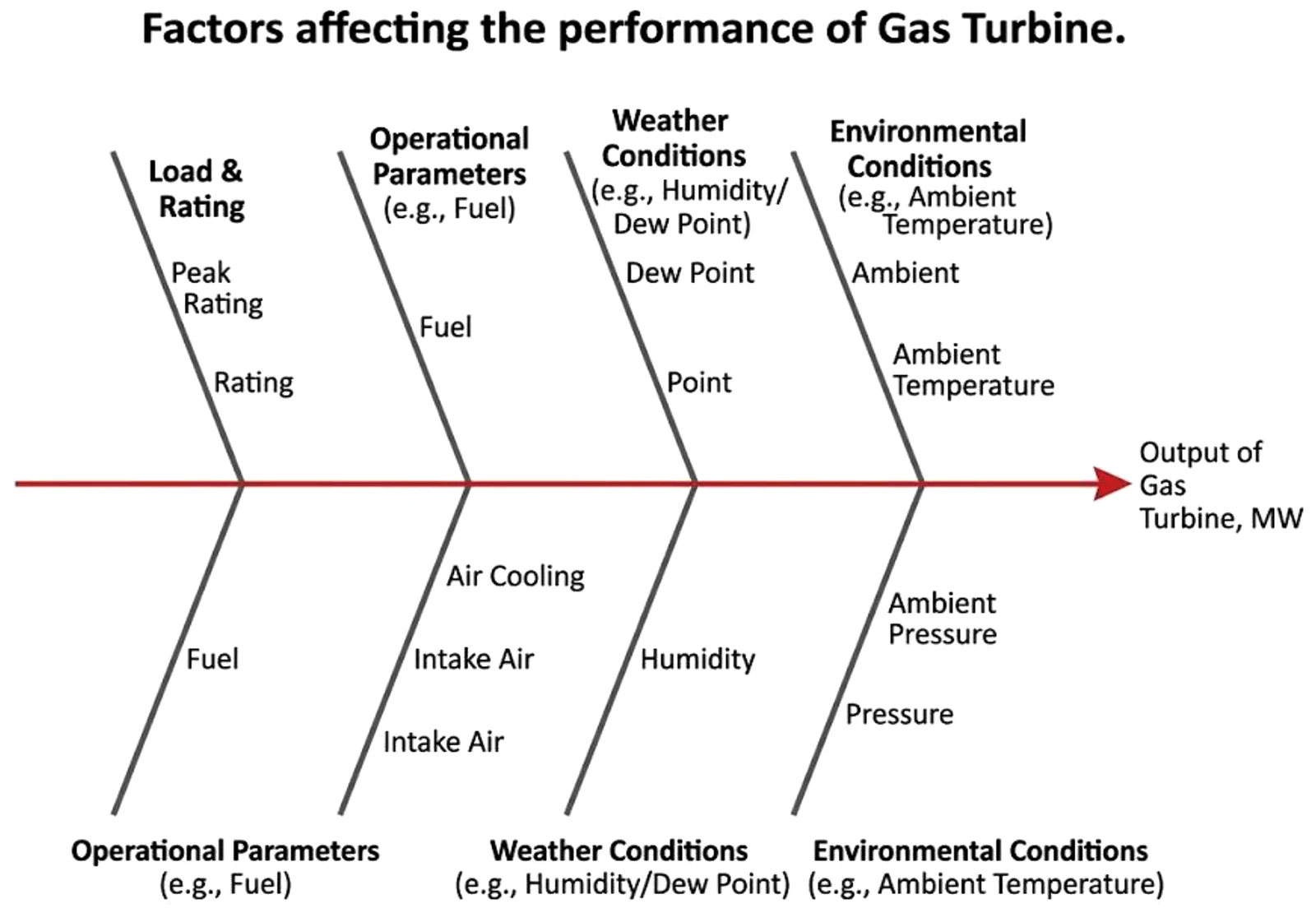
Parameters affecting the output of Gas Turbine GT.

Nandipur power plant is dual fuel and can work on liquid fuel (HSD, HSFO) and on gas fuel as well. After hot gas path inspection and conversion to gas back in 2017. Currently, Nandipur power plant is working on gas fuel with its increased capacity of 525 MW with more reliability and lifecycle. Nandipur at its base load has a capacity to generate 12.6 million units per day and 378 million units per month. Quality and maintenance of the turbine is monitored continuously and further the maximum capacity of turbine is defined after monitoring quality of turbine every year. Multiple parameters play a vital role on the performance of gas turbine. Cause and effect diagram is shown in Fig 1. It can be noted that majorly ambient temperature, dew point, humidity, ambient pressure, fuel, water injection, air intake cooling and peak rating are the major factors affecting the performance of power generation. For this study dewpoint, humidity and ambient temperature are selected for detailed analysis and discussion. It was noted that ambient pressure remains almost constant. Data has been collected at maximum base load. All other values when base load is not maximum has been ignored.

Multiple devices have been used for data collection in this stud. Different parameters are effecting turbine as shown in Fig 1. Ambient temperature in °C has been calculated by 96 HR. Similarly, dew point in °C has been observed with dew point meter (96HR). Generation power in megawatt (MW) has been observed with digital multimeter (DMM). Reactive power in KVA values also collected from DMM. Compressor discharge pressure in Bar was calculated from 96 CD. Ambient pressure in mmHg has been observed with 96AS.

To conduct this study data has been collected for one year from real running system. Data has been collected from 3 turbines from 6th September-2021–2nd September-2022. Parameter noted after every 1 hour by making sure that gas turbine is on baseload (full load), while if gas turbine is on preselect load (Partial Load) or standstill then no data taken or considered. As in this project our focus is to predict the maximum performance of gas turbine so only data on base load was considered and further processed. Data collection is one of the critical tasks in this project. Around 2 months are utilized for data validation and convert it to Excel file and reverification of data. Data is re-verified by another author to enhance the validity of data. Details of data are given in Table 1.

**Table 1:** Descriptive Statistics.

Descriptive Statistics						
	Gas Turbine 1		Gas Turbine 2		Gas Turbine 3	
Variables	Mean	Std Dev	Mean	Std Dev	Mean	Std Dev
Ambient Temperature (°C)	32.816	5.176318	32.478	5.284573	33.06	5.438463
Generation Active Power (MW)	105.486	4.335397	105.885	4.776428	107.23	4.750115
Reactive Power (MW)	27.896	16.0915	23.458	17.56269	24.85	17.81352
Apparent Power (KVA)	110.28	4.534373	109.86	4.884693	111.51	4.553443
Compressor Discharge Pressure (bar)	10.128	0.231389	10.16	0.255831	10.17	0.276742
Ambient Pressure (mmHG)	730.99	16.19792	730.94	15.36202	730.87	17.05659
Dew Point (°C)	18.55096	6.415315	17.98075	6.477831	17.47017	6.695517
Humidity (%)	48.41204	21.17998	47.54252	21.03054	44.87184	20.88528

Humidity has been calculated from Equation 1. Where, DP is dew point, AT is ambient temperature.


$$\begin{array}{c} Humidity=\text{1}\text{0}\text{0}\;\times \left(\frac{\text{EXP }\left(\frac{\text{1}\text{7}.\text{6}\text{2}\text{5}\;\times \text{DP}}{\text{2}\text{4}\text{3}.\text{0}\text{4}+\text{DP}}\right)}{\text{EXP }\left(\frac{\text{1}\text{7}.\text{6}\text{2}\text{5}\;\times \text{AT}}{\text{2}\text{4}\text{3}.\text{0}\text{4}+\text{AT}}\right)}\right) \end{array}$$ (1)

Furthermore, in this study apparent power also has been calculated from Equation 2. Where AP is the active power and RP is the reactive power.


$$\begin{array}{c} \text{Apparent}\;\text{Power}=\sqrt{(AP{)}^{\text{2}}}+(\text{RP}{)}^{\text{2}} \end{array}$$ (2)

From gas turbine 1 ambient temperature, generation active power, active power, apparent power, and compressor discharge pressure data have been collected. Total 1566 values are noted. Ambient temperature (AT) is noted as 32.8160 ± 5.1763 °C. Active power (AP) of 105.486 ± 4.335 MW and reactive power (RP) of 27.869 ± 16.09 MW. The apparent power is calculated as 110.28 ± 4.5343 KVA using equation 2. Compressor discharge pressure of 10.128 ± 0.2313 bar and ambient pressure of 730.99 ± 16.19 mmHG are also measured. Dew Point of 18.5509 ± 6.415 was noted that helps to calculate the humidity of 48.412 ± 21.17 using equation 1.

From Gas Turbine II total 1704 values are noted. The ambient temperature of 32.478 ± 5.284°C is recorded. Generation MW active power of 105.885 ± 4.776 MW and reactive power of 23.485 ± 17.56MW lead to calculation of apparent power of 109.86 ± 4.88 KVA. Compressor discharge pressure of 10.160 ± 0.255 bar and ambient temperature of 730.94 ± 15.36 mmHG were noted. At the dew point of 17.98 ± 6.477, the humidity of 47.542 ± 21.03 was measured using equation 1.

From Gas Turbine III total 1438 values are noted. The ambient temperature of 33.06 ± 5.4384°C and generation MW active power of 107.23 ± 4.75 MW align with reactive power of 24.85 ± 17.813MW are noted. The apparent power of 111.51 ± 4.55 KVA is obtained at compressor discharge pressure of 10.17 ± 0.276 bar. The ambient temperature of 730.87 ± 17.05 mmHG and dew point of 17.47 ± 6.69 lead to calculation of humidity of 44.87 ± 20.88 using equation 1.

**Fig 2:**
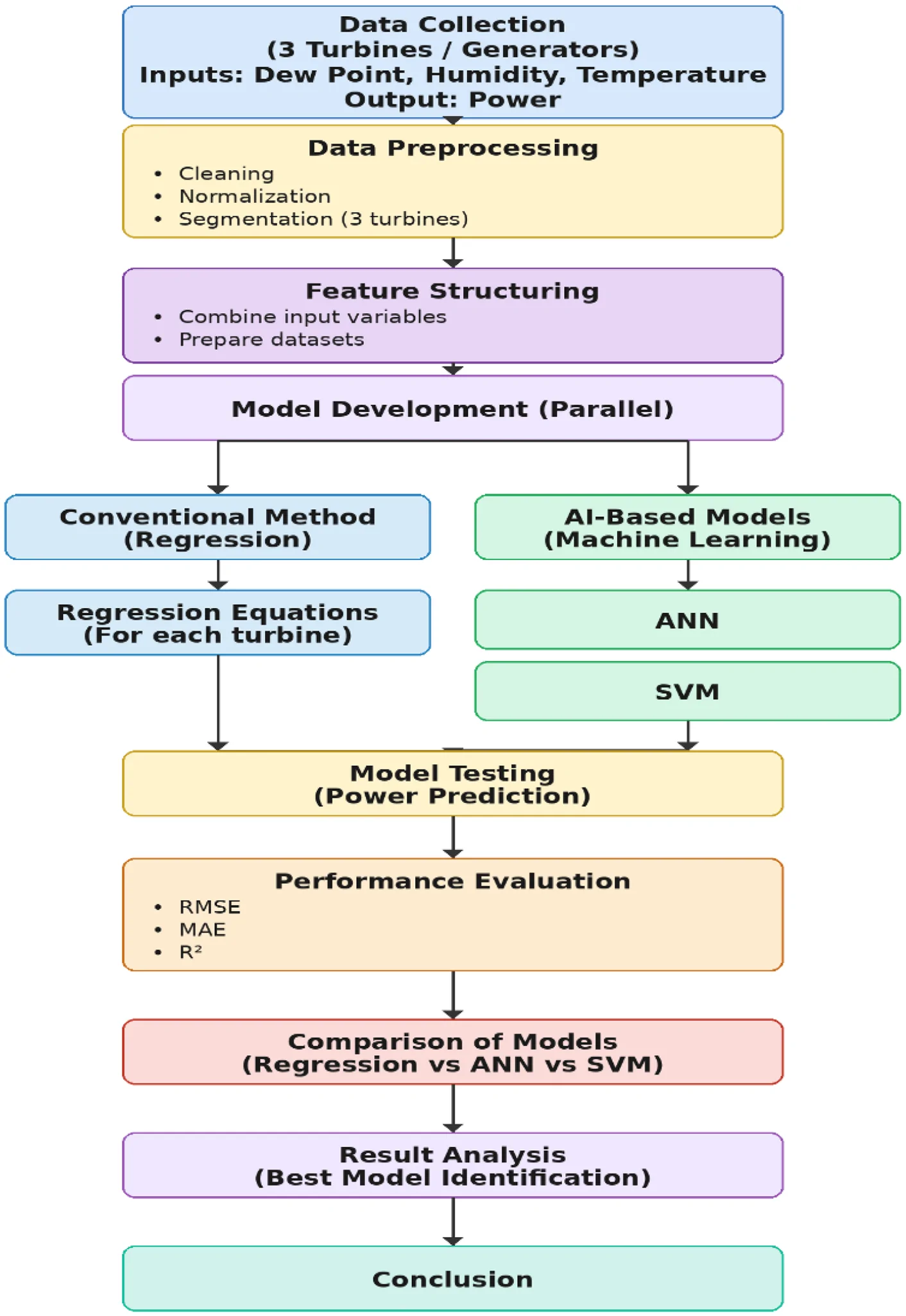
Important steps of Methodology.

This study consists of multiple steps as shown in Fig 2. First step is real-time data collection from site and its verification. To develop deep familiarization with the collected data, descriptive analysis technique was applied. To further analyze the data, correlations have been found. Correlation has been found between: 1) Power and ambient temperature, 2) Power and dew point, 3) Power and humidity. As data was collected from three gas turbines so correlation is analyzed for all three separately. The correlation data is further analyzed using a regression equation. In this regard, a regression equation calculator is introduced in this study. Finally, verification of regression equation is performed based upon the calculation of percentage error. Percentage error is calculated from Equation 3. Where OV is the original value and PV is the predicted value. Minitab has been used for analysis of data. A calculator is designed upon regression equation so we can predict the performance of gas turbine. In this way calculated active power has been validated by the active power measured by DMM.


$$\begin{array}{c} \%\text{ ERROR }=\;\left(\frac{OV-PV}{OV}\right)\times \text{1}\text{0}\text{0} \end{array}$$ (3)

Simple regression has been adopted to predict the performance of gas turbines. Regression equation is one of the extensively used and reliable techniques specially used for prediction purposes.

### Linear regression

Linear regression is one of the methods that have been frequently implemented in the past for determining the relationship between gas turbine power production and the ambient conditions of the location [18,19,47]. This paper considers the use of multiple linear regression for linking a dependent variable (active power output, P), with three independent ambient variables namely ambient temperature (T), dew point (DP), and relative humidity (RH). Multiple linear regression model is generally given by this equation 4.


$$\begin{array}{c} P=\beta_{\text{0}+}\beta_{\text{1}}T+\beta_{\text{2}}DP+\beta_{\text{3}}RH+\varepsilon \end{array}$$ (4)

Here, P stands for the active power output (in MW) predicted by the model; β_0_ corresponds to the intercept (bias term); β_1_, β_2_, and β_3_ represent the regression coefficients for ambient temperature, dew point, and relative humidity, respectively; and is the residual error. Estimated regression coefficients rely on the ordinary least squares method that aims to minimize the sum of the squared residuals between the actual and fitted values [19] as given in equation 5.


$$\begin{array}{c} minimize\;\sum_{i}{\left(P_{i}-{\text{P}\hat{\;}}_{i}\right)}^{\text{2}} \end{array}$$ (5)

Where Pᵢ is the actual power output and P̂ᵢ is the predicted power output obtained from the model for the i-th observation.

To measure the degree of linear association between each of the ambient variables and the active power output, the calculation of the Pearson correlation coefficient (r) was done [43,45] and shown in equation 6


$$\begin{array}{c} =\frac{\sum \left(x_{i}-\overset{―}{x})(y_{i}-\overset{―}{y}\right)}{\sqrt{\left[\sum {\left(x_{i}-\overset{―}{x}\right)}^{\text{2}}.\sum {\left(y_{i}-\overset{―}{y}\right)}^{\text{2}}\right]}} \end{array}$$ (6)

Here, xᵢ and yᵢ are the individual samples of the ambient variable and active power output respectively, while x̄ and ȳ represent their respective means. The value of r lies between −1 and +1, with values closer to ±1 implying a stronger linear relationship.

Furthermore different machine learning algorithms trained through MATLAB Regression Learner App (MathWorks Inc.) [15,17]. For both SVM and ANN algorithms 5-fold cross-validation has been implemented. The prediction accuracy of the models was quantified using performance metrics, including coefficient of determination (R^2^), root mean square error (RMSE), and mean absolute error (MAE). For all machine learning algorithms the three ambient variables — ambient temperature, dew point, and relative humidity — were used as input features, while the active power output was the target output variable. All machine learning algorithms have been separately trained and validated for each of the data obtained from GTI, GTII and GTIII.

### Support vector machine

Five SVM models were implemented using different kernel functions: Linear, Quadratic, Cubic, Fine Gaussian, and Medium Gaussian. The Linear, Quadratic, and Cubic SVM models employed their respective polynomial kernel functions, with all remaining optimization parameters set to auto to allow the solver to determine optimal settings automatically. The Fine Gaussian and Medium Gaussian models utilized the Gaussian radial basis function kernel, with kernel scale values manually set to 0.75 and 3.0 respectively, controlling the locality of the kernel’s influence on the decision boundary — a smaller kernel scale in the Fine Gaussian model produces a more localized fit, while the larger kernel scale in the Medium Gaussian model yields a smoother and more generalized prediction surface. The box constraint and other hyperparameters for all models were optimized through cross-validation on the training dataset.

SVM regression is one of the most effective regression techniques which have competitive predictive power for gas turbine and energy system applications. Its effectiveness is in particular proven in setup with medium-sized datasets capable of handling nonlinear relations between input-output variables [22,23]. In the setup of SVM, the input vector x = [T, DP, RH]^T^ is first projected to a high-dimensional space using kernel function. After that, the SVM proposes a regression model f(x) which calculates the output within maximum deviation ε from the real point while the function f(x) remains as simple as possible. The overall support vector regression function is given in equation 7.


$$\begin{array}{c} f(x)=\omega^{T}\Phi (x)\;+\;b \end{array}$$ (7)

Here w is the weight vector, φ(x) represents the feature map, and b is the bias term. The best choice of w and b is obtained after solving this optimization problem with constraints [22,23] and shown in equation 8.


$$\begin{array}{c} minimize\;\frac{\text{1}}{\text{2}}{\left\|\omega \right\|}^{\text{2}}+C.\sum_{i}(\xi_{i}+\xi_{i}^{*}) \end{array}$$ (8)

subject to equation 9–11.


$$\begin{array}{c} y_{i}-\omega^{T}\varphi (\chi_{i})-b\leq \;\varepsilon \;+\;\xi_{i} \end{array}$$ (9)

$$\begin{array}{c} \;\omega^{T}\varphi (\chi_{i})\;+b\;-\;y_{i}\leq \;\varepsilon \;+\;\xi_{i}^{*} \end{array}$$ (10)

$$\begin{array}{c} \xi_{i}{,\xi }_{i}^{*}\geq 0 \end{array}$$ (11)

Where C is the regularization parameter controlling the model flatness and the tolerance to those deviations that extend ε; ξᵢ and ξᵢ* slack variables allowing the errors that go beyond the -insensitive zone. In this work, six kernel functions were used, defining different mappings φ(x) [13,23]. Different Kernels used in this study are given in equation 12–15.

**Linear Kernel:**

$$\begin{array}{c} \;K(\chi_{i}\;\chi_{j})=\chi_{i}^{T}\chi_{j} \end{array}$$ (12)

**Quadratic Kernel:**

$$\begin{array}{c} K(\chi_{i}\;\chi_{j})={{(\chi }_{i}^{T}\chi_{j\;}+\;\text{1})}^{\text{2}} \end{array}$$ (13)

**Cubic Kernel:**

$$\begin{array}{c} K(\chi_{i}\;\chi_{j})={{(\chi }_{i}^{T}\chi_{j\;}+\;\text{1})}^{\text{3}} \end{array}$$ (14)

**Fine Gaussian Kernel (σ = σ_fine):**

$$\begin{array}{c} K(\chi_{i}\;\chi_{j})=exp(\frac{-{\left\|\chi_{i}-\chi_{j}\right\|}^{\text{2}}}{\text{2}\;\sigma^{\text{2}}}) \end{array}$$ (15)

σ – the kernel width parameter. The difference among Fine, Medium and Coarse Gaussian kernels lies in the value of σ which is smaller for Fine, leading to more local, sensitive fit to data variations and larger for Coarse, resulting in a fitter that is smoother and more generalized [23]. Choosing the best kernel and setting the hyperparameters were done based on cross-validation results on the training data.

### Artificial neural network

In this study, five ANN architectures were investigated: Narrow, Medium, Wide, Bilayer, and Trilayer. All architectures employed the Rectified Linear Unit (ReLU) as the activation function in the hidden layers, with a maximum iteration limit of 1000 to ensure sufficient convergence during training. The Narrow, Medium, and Wide architectures each consist of a single hidden layer containing 10, 25, and 100 neurons respectively, providing varying levels of model complexity within a shallow network structure. The Bilayer architecture comprises two hidden layers, while the Trilayer architecture comprises three hidden layers, each containing 10 neurons per layer, enabling hierarchical feature extraction from the input ambient variables. The number of input neurons corresponded to the three ambient input variables — ambient temperature, dew point, and relative humidity — while the output layer consisted of a single neuron representing the predicted active power output.

ANNs are a very well-established tool in the field of gas turbine power and thermal performance prediction [20,21,29,47]. Given an input vector x = [T, DP, RH]^T^, an ANN executes the mapping to output active power P through a chain of connected layers. Each layer consists of neurons operating a weighted summation transformation followed by a nonlinear activation function given in equation 16.


$$\begin{array}{c} \alpha_{j}^{\left(l\right)}=\;f(\sum_{k}{\text{w}}_{jk}^{l}.a_{k}^{\left(l-\text{1}\right)}+b_{j}^{\left(l\right)}) \end{array}$$ (16)

Here, w_jk_^(1)^ defines the weight of the link between neuron k of layer (l − 1) and neuron j of layer l; b_j_^(1)^ is the bias of the neuron j in layer l; a is the output of the neuron k from the previous layer, and f(.) is the nonlinear activation function. The hidden layers used sigmoid activation functions [29] given in equation 17.


$$\begin{array}{c} f(z)\;=\;\frac{\text{1}}{\text{1}+e^{-z}} \end{array}$$ (17)

The layer at the end has a linear activation function to generate continuous power output predictions. In this study, five neural network architectures differing in the number of hidden layers and number of neurons per layer were implemented [20,47].

#### Narrow neural network.

One hidden layer with a small number of neurons (10 neurons), good for capturing simple nonlinear relationships with low chance of overfitting.

#### Medium neural network.

One hidden layer with an intermediate number of neurons (25 neurons), this number strikes a balance between model complexity and preventing overfitting.

#### Wide neural network.

One hidden layer, but with a large number of neurons (100 neurons), this network Because of this has a high capacity to represent complex nonlinear data.

#### Bilayer neural network.

Two hidden layers, each layer with 10 neurons, allows the neural network to understand hierarchical features from input variables.

#### Trilayer neural network.

Three hidden layers, each layer having 10 neurons Though the training becomes more difficult.

Levenberg-Marquardt backpropagation algorithm was employed for training all these network architectures. This method updates weights and biases in iterations to reduce the mean squared error between predicted and observed power output [21,29] given in equation 18.


$$\begin{array}{c} MSE\;=\;\frac{\sum_{i}{{(P}_{i}-{\text{P}\hat{\;}}_{i})}^{\text{2}}}{n} \end{array}$$ (18)

Following the data partition method of the gas turbine neural network studies [21,29], a randomized split was conducted to create the training, validation, and testing subsets respectively.

### Model evaluation metrics

The predictive accuracy of all regression, SVM, and ANN models was most crucially evaluated by three performance metrics that are widely used and standard in the fields of gas turbine and energy system prediction studies [19,23,43]. Root mean square error measures the average size of the prediction error roughly in the same units as the output variable given in equation 19.


$$\begin{array}{c} RMSE\;=\;\sqrt{\left[\frac{\sum_{i}{{(P}_{i}-{\text{P}\hat{\;}}_{i})}^{\text{2}}}{n}\right]} \end{array}$$ (19)

Meanwhile mean absolute error is a linear measure of the average absolute prediction deviation given in equation 20.


$$\begin{array}{c} MAE\;=\;\frac{\sum_{i}\left|P_{i}-{\text{P}\hat{\;}}_{i}\right|}{n} \end{array}$$ (20)

At last, the coefficient of determination (R^2^) represents what fraction of the variance in the observed active power output is explained by the model [19,20,23] as given in equation.


$$\begin{array}{c} R^{\text{2}}=\;\text{1}-\;\frac{\sum_{i}{\left(P_{i}-{\text{P}\hat{\;}}_{i}\right)}^{\text{2}}}{\sum_{i}{\left(P_{i}-\overset{―}{p}\right)}^{\text{2}}} \end{array}$$ (21)

In this equation, P̄ denotes the mean of observed active power values, n stands for the total number of observations, P̂ᵢ is the observed value, and P̂ᵢ is the model-predicted one. R^2^ equal to 1. 0 means a perfect fit, whereas R^2^ values that happen to be closer to 0 imply the model explains the variation poorly. As a result, the three metrics were separately calculated for GT I, GT II, and GT III to facilitate the cross-unit comparison of the quality of the modeling.

## Results

**Fig 3:**
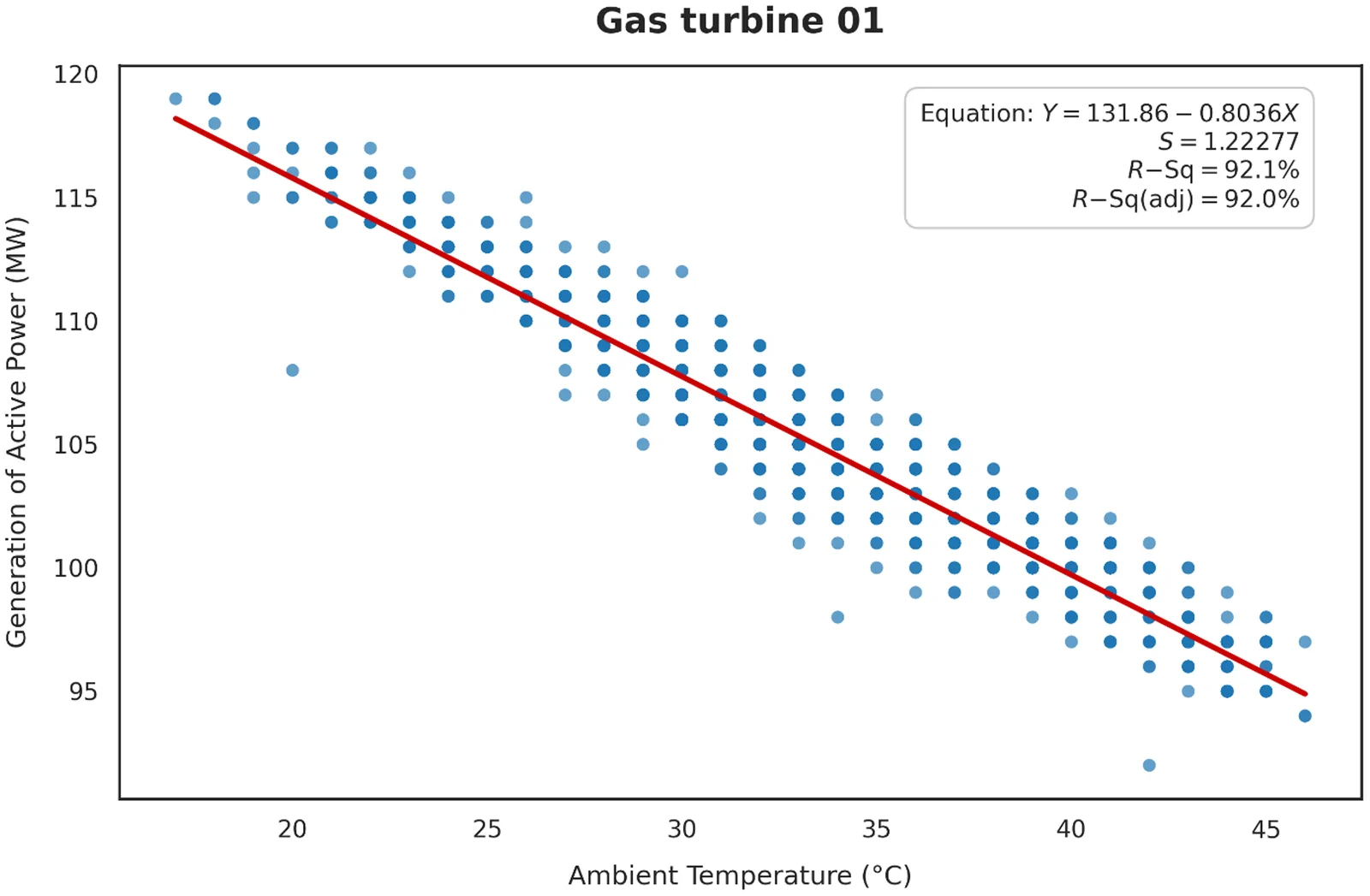
Correlation between apparent power and ambient temperature for Gas turbine 1.

**Fig 4:**
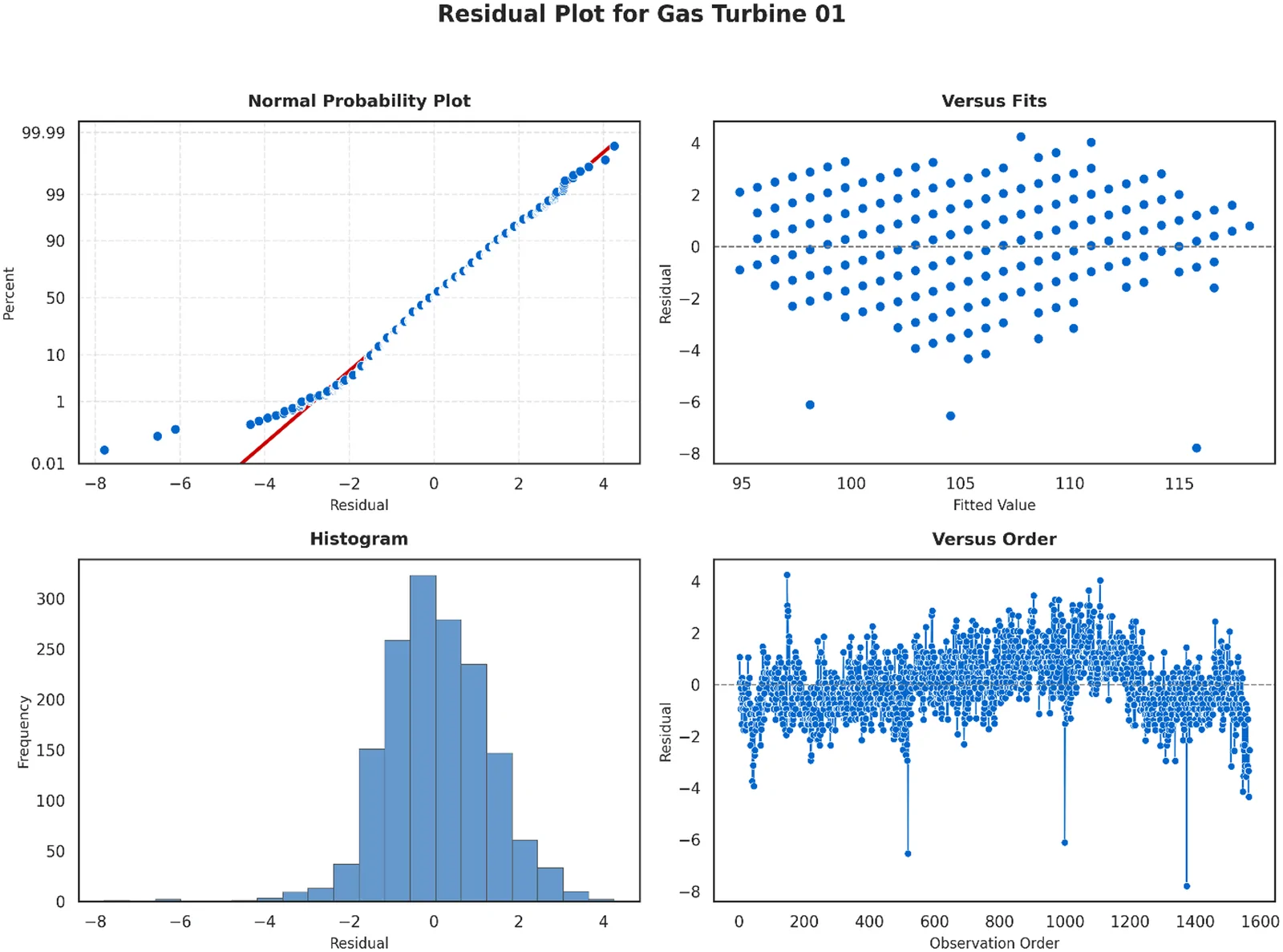
Residual plot between apparent power and ambient temperature for Gas turbine 1.

The correlation of ambient temperature and active power indicated r = −0.959, R^2^ = 92.1%, Adjusted R^2^ = 92.0%, S = 1.22. Obtained values exhibit a strong negative linear relationship between both variables. Over 92% of the variation in active power generation is explained by ambient temperature. The low standard error indicates high model precision. This regression is highly suitable for predictive analysis and operational control. From the graphs in Figs 3 and 4 it’s easy to see that there is a strong and clear negative relationship between ambient temperature and the generation of active power. As the temperature increases, the active power noticeably decreases. This can also be seen from the trend line that slopes (Fig 4). The points are closely packed around the line, which means the data is quite consistent and the relation is strong. Furthermore, correlation has been identified for GT II and GT III correlation is r_2_ = −0.8256 and r_3_ = −0.9472 with ambient temperature which indicates strong relation.

**Fig 5:**
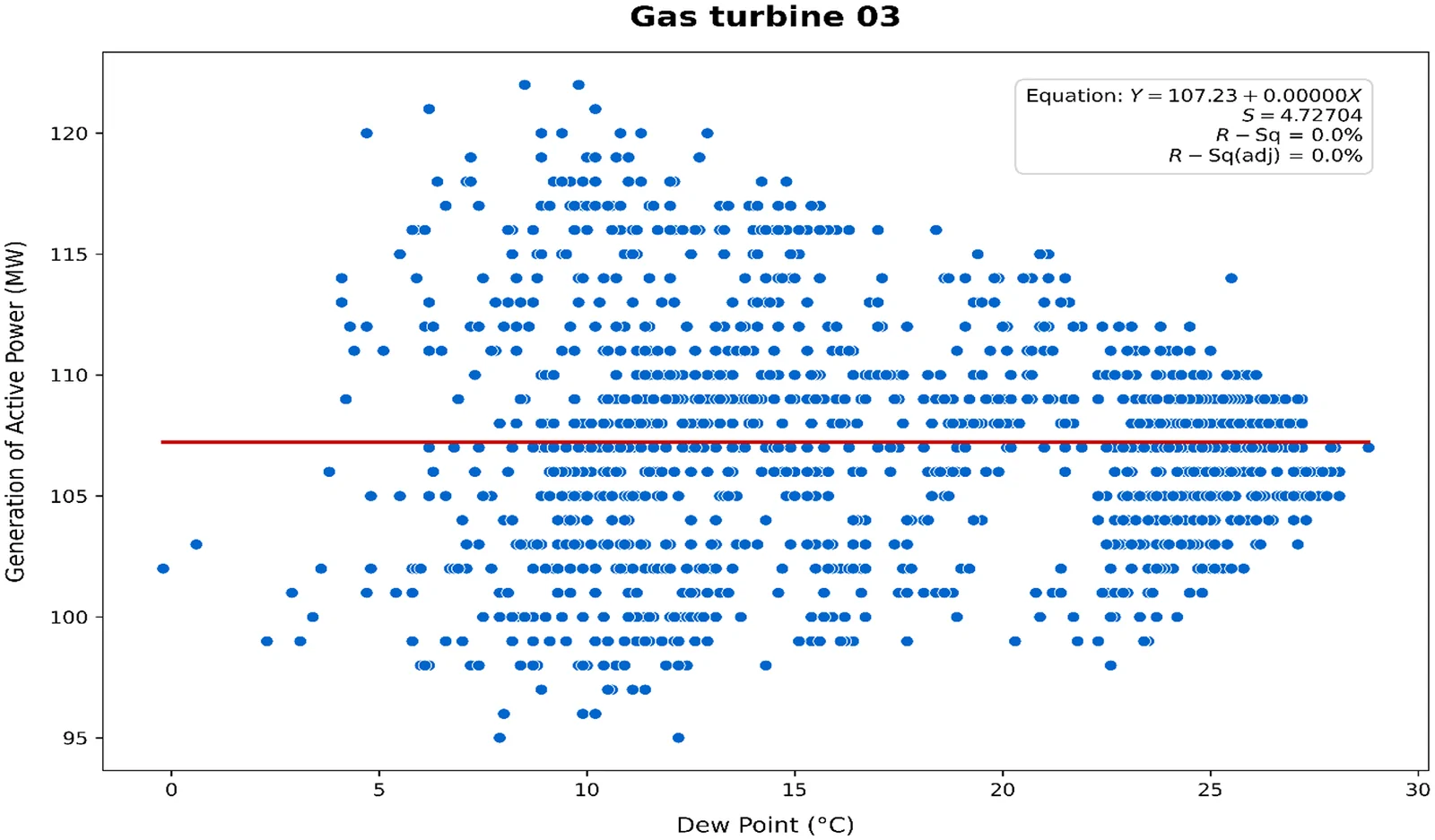
Correlation between apparent power and dew point for gas turbine 3.

**Fig 6:**
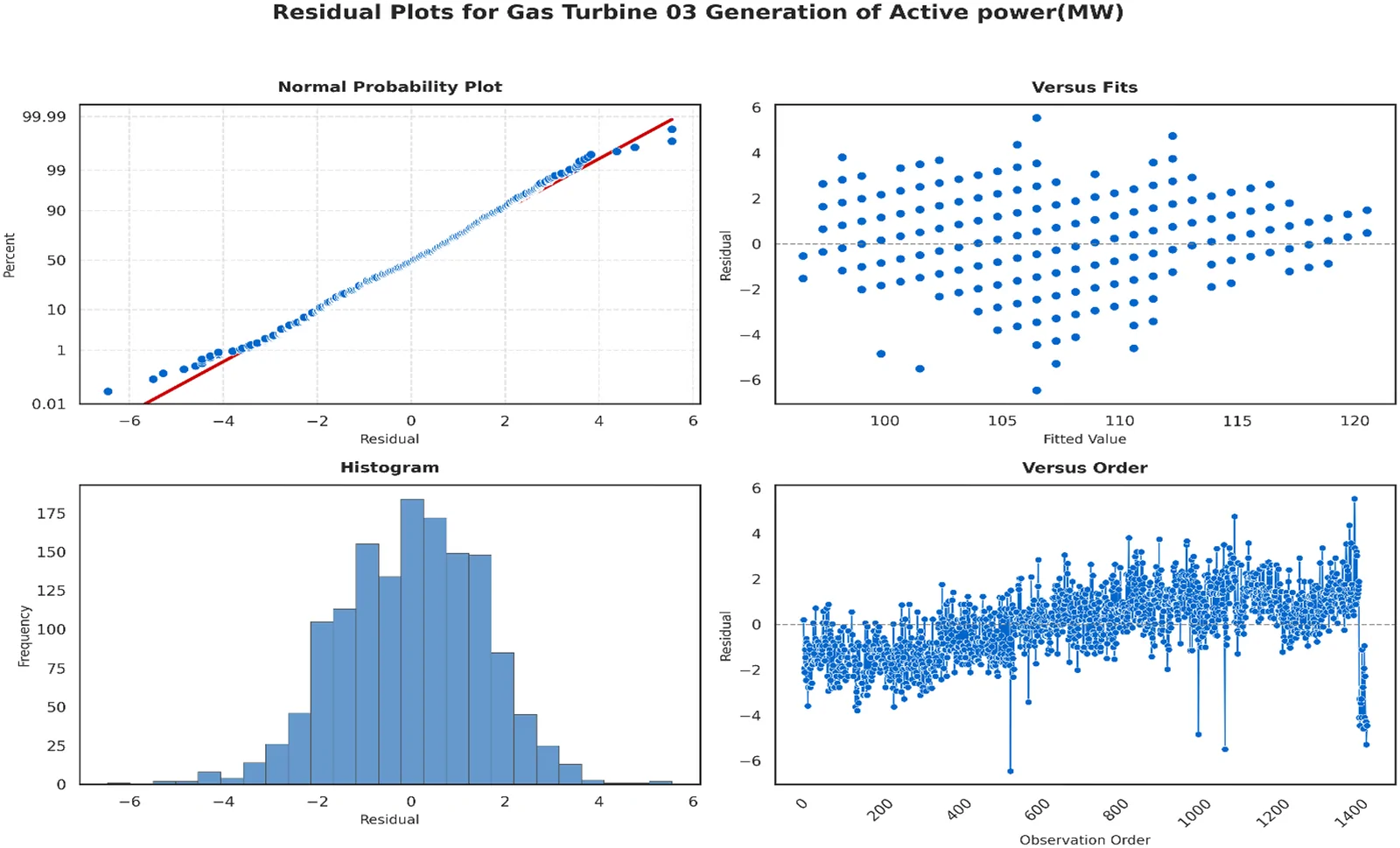
Residual plot between apparent power and dewpoint for gas turbine 3.

The correlation of dew point and active power indicated r = −0.0631, R^2^ = 0.4%, Adjusted R^2^ = 0.3%, S = 4.32 (Figs 5 and 6). Obtained values exhibits no relationship between both variables. This regression is not suitable for predictive analysis and operational control. In Figs 5 and 6, there is a week relationship and points are loosely packed around the line. Furthermore, correlation has been identified for GT II and GT III correlation is r_2_ = −0.0079 and r_3_ = −0.1019 with dew point.

**Fig 7:**
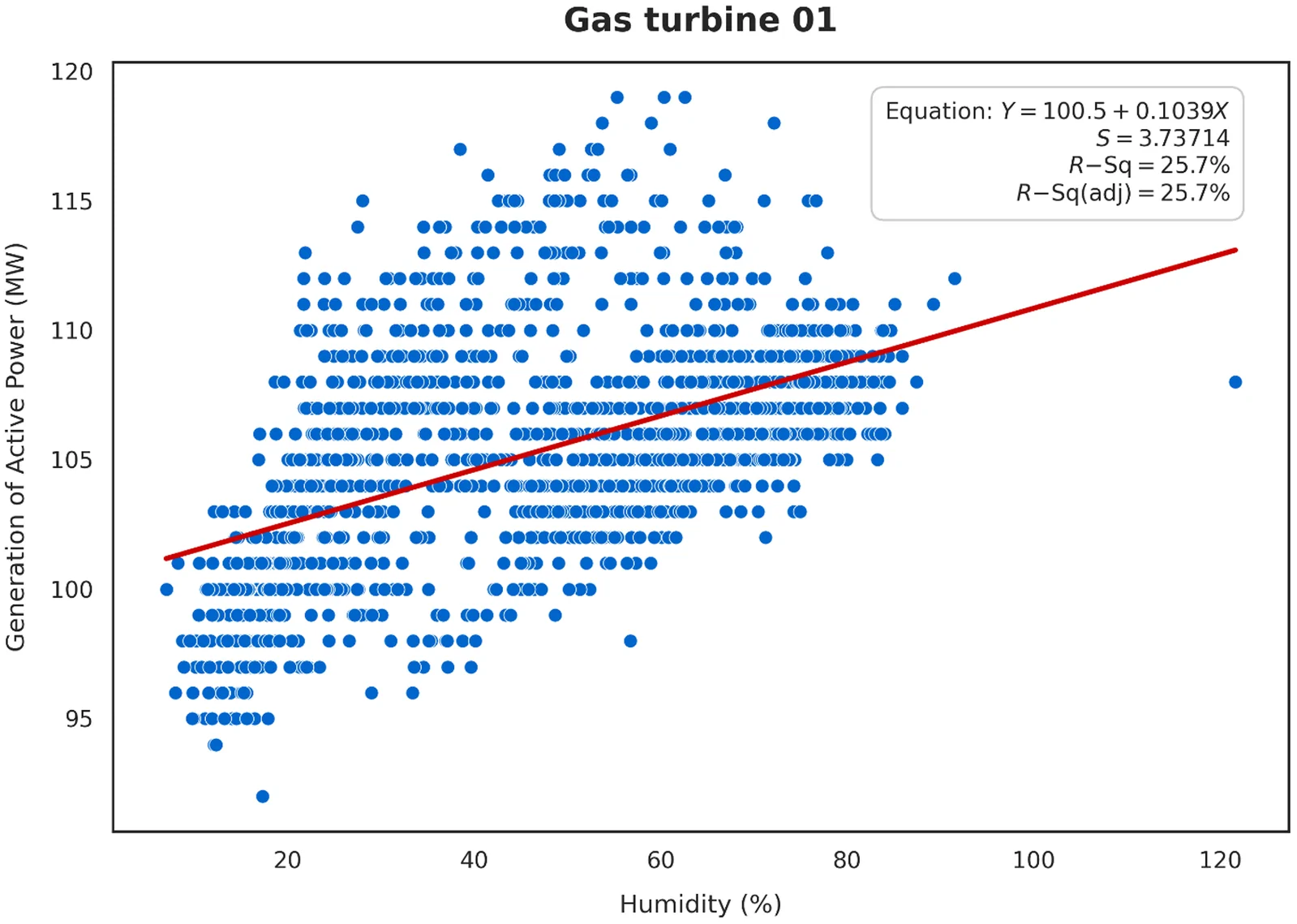
Correlation between apparent power and Humidity for gas turbine 1.

**Fig 8:**
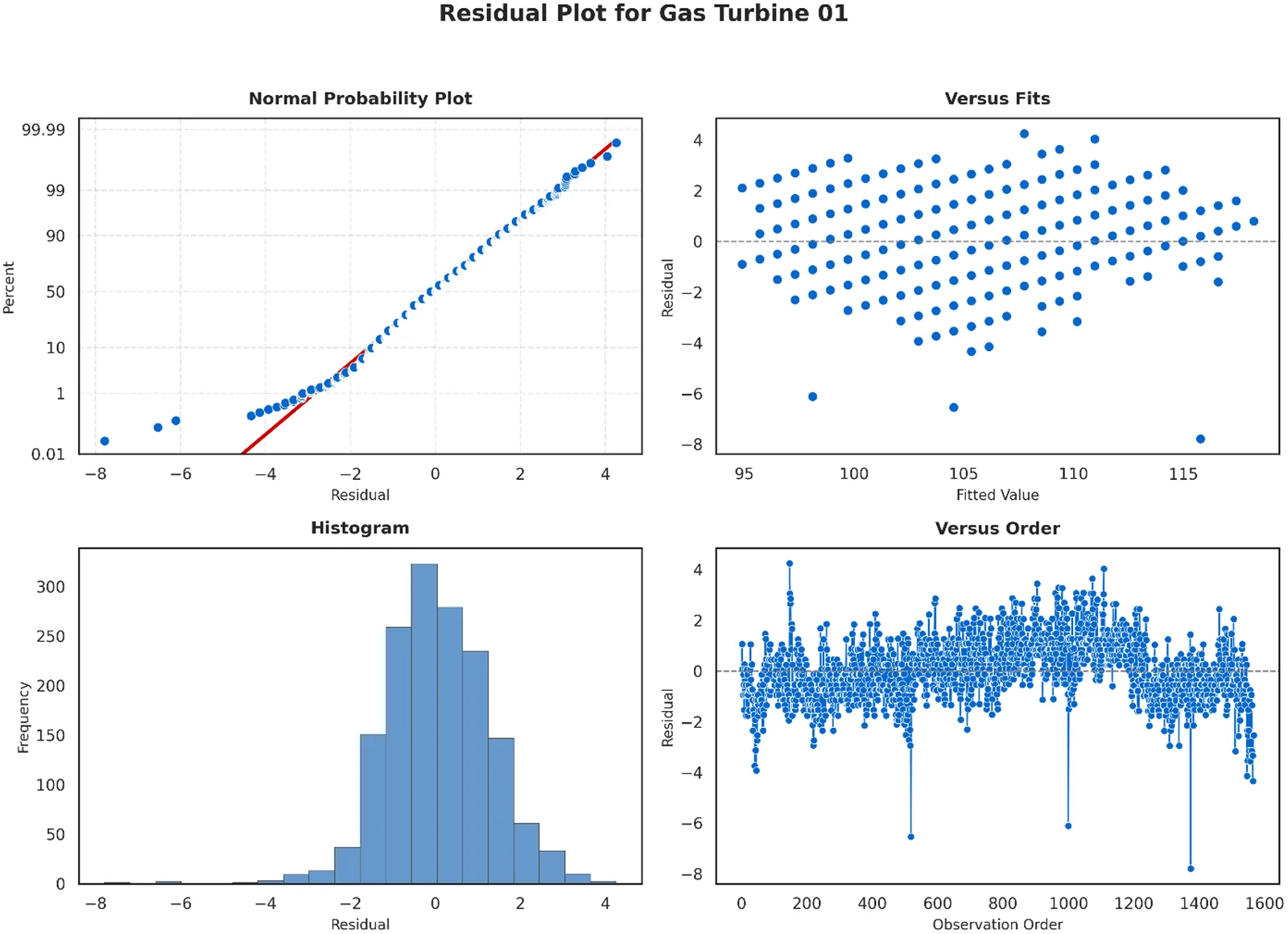
Residual plot between apparent power and dew point for gas turbine 1.

The correlation of humidity and active power indicated r = 0.5074 R^2^ = 25.7%, Adjusted R^2^ = 25.7%, S = 3.73 (Figs 7 & 8). Obtained values exhibit weak-to-moderate relationships between both variables. Over 26% of the variation in active power generation is explained by ambient temperature. While the standard error is relatively lower, the explanatory power is still limited. The model may have some supporting role in multivariate analysis but is insufficient. This regression is not highly suitable for predictive analysis and operational control. In Figs 7 & 8, there’s weak-to-moderate relationship between humidity and the generation of active power. The points are not closely packed around the line, which means the data is not consistent and the relation is moderate. The correlation for GT II and GT III is found as r_2_ = 0.4964 and r_3_ = 0.4752 with humidity.

Correlation of ambient pressure with apparent power is calculated as shown in Table 2. It can be noticed that ambient pressure also indicates weak correlation. For gas turbine GT I, GT II and GT III, the respective correlations found are r_1_ = 0.171, r_2_ = 0.1486 and r_3_ = 0.178 respectively with ambient pressure.

**Table 2:** Results of Conventional method.

Correlation	Ambient Temperature (°C)	Dew Point (°C)	Humidity (%)	Ambient Pressure of mmHg
GT I Active Power (MW)	−0.9594	0.0631	0.5074	0.1711
GT II Active Power (MW)	−0.8256	−0.0079	0.4964	0.1486
GT III Active Power (MW)	−0.9472	−0.1019	0.4752	0.1782

From correlation it can be noted that ambient temperature has indicated strong relation with power. In this way for Regression technique, only one factor of ambient temperature is considered. Therefore, in regression analysis, ambient temperature is considered as independent variable and gas turbine Active power as dependent variable in order to find regression equation for gas turbine # I, II, III based upon the previous one-year data that was collected when gas turbine generating its energy at its base load. Regression Equations 22, 23 and 24 have been developed to calculate apparent Power (AP_1,_ AP_2_, AP_3_) from ambient temperature in °C has been calculated for gas turbine I, II, and III.


$$\begin{array}{c} \text{AP1}=\text{1}\text{3}\text{1}.\text{8}\text{5}\text{6}-(\text{0}.\text{8}\text{0}\text{3}\text{5}\text{6}\times \text{AT}) \end{array}$$ (22)

$$\begin{array}{c} \text{AP2}=\text{1}\text{3}\text{0}.\text{1}\text{2}\text{1}-(\text{0}.\text{7}\text{4}\text{6}\text{2}\times \text{AT}) \end{array}$$ (23)

$$\begin{array}{c} \text{AP3}=\text{1}\text{3}\text{4}.\text{5}\text{8}\text{0}-(\text{0}.\text{8}\text{2}\text{7}\text{2}\text{7}\times \text{AT}) \end{array}$$ (24)

**Fig 9:**
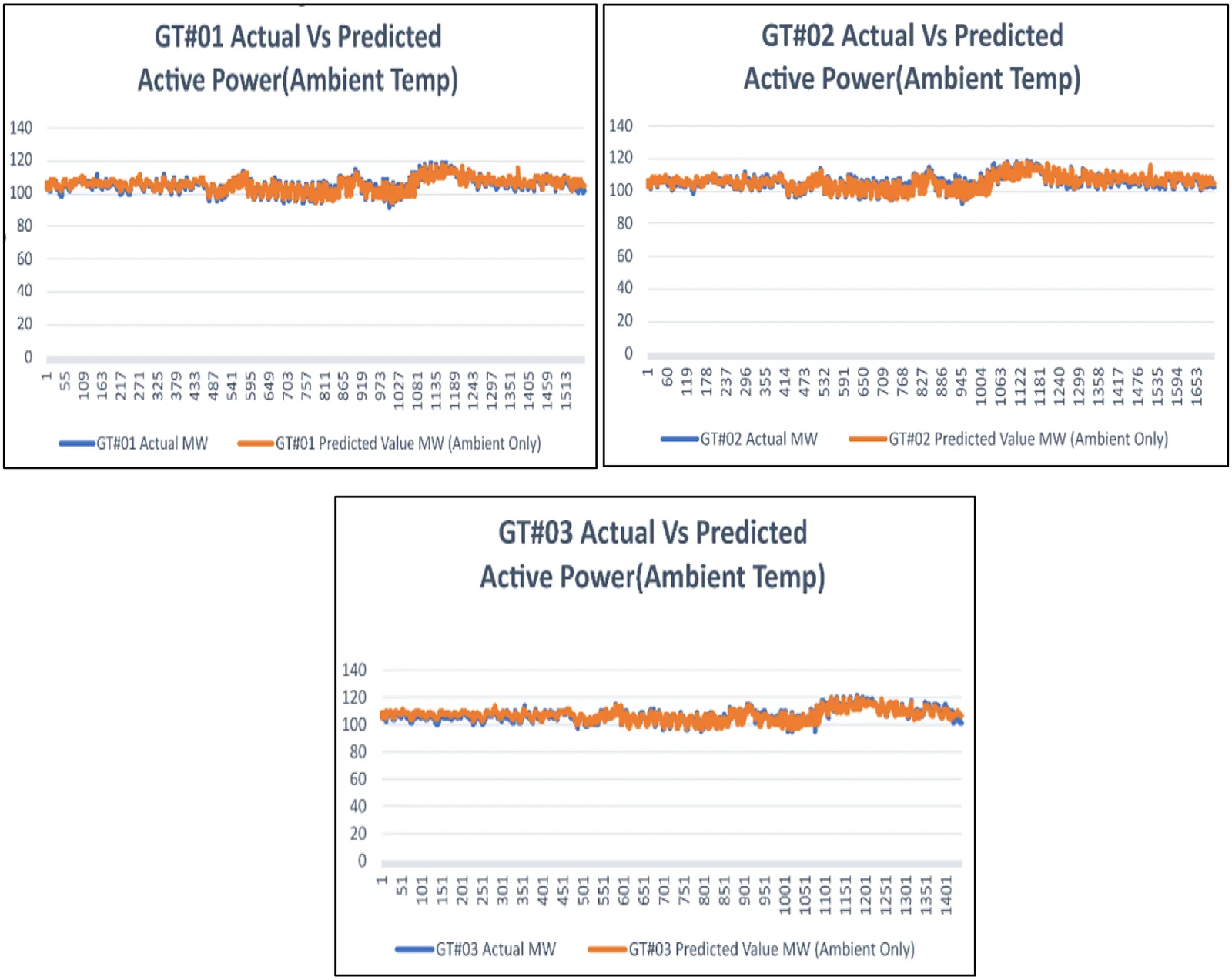
Validation with error trends of Gas Turbine I, II and III.

After getting regression equation, in order to verify the reliability of simple regression equation based upon ambient temperature, same equation also implemented to real data. The analysis is again performed to find error based upon predicted value and real value. The values of the error for GT# I, II, III are respectively calculated as [Error = −0.01088, −0.53353, −0.01182]. The error trend is illustrated in Fig 9. It shows the comparison between actual versus predicted. The predicted values are estimated from the proposed.

### AI- based analysis

**Fig 10:**
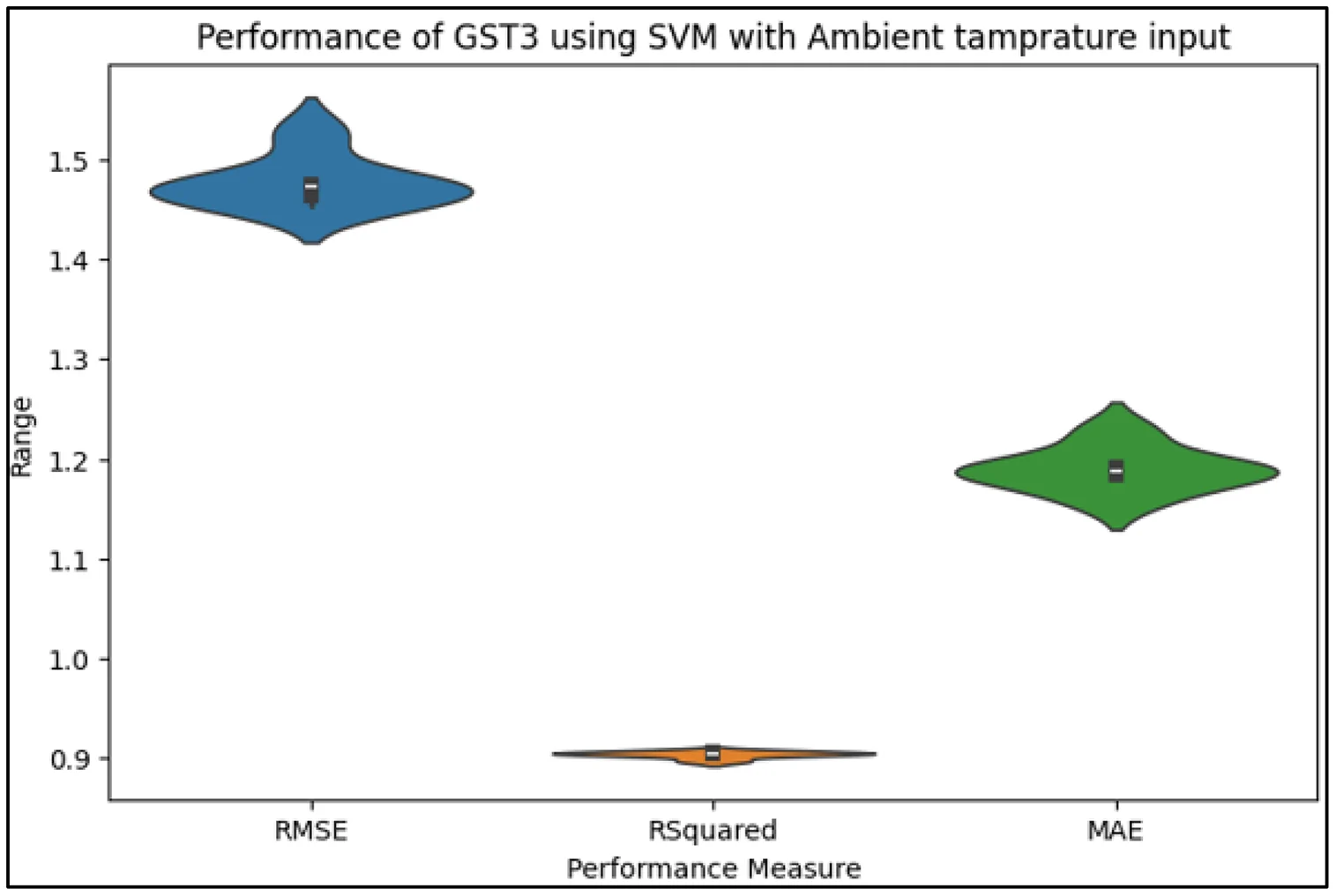
Result of SVM classifier using Ambient temperature as input parameter.

**Fig 11:**
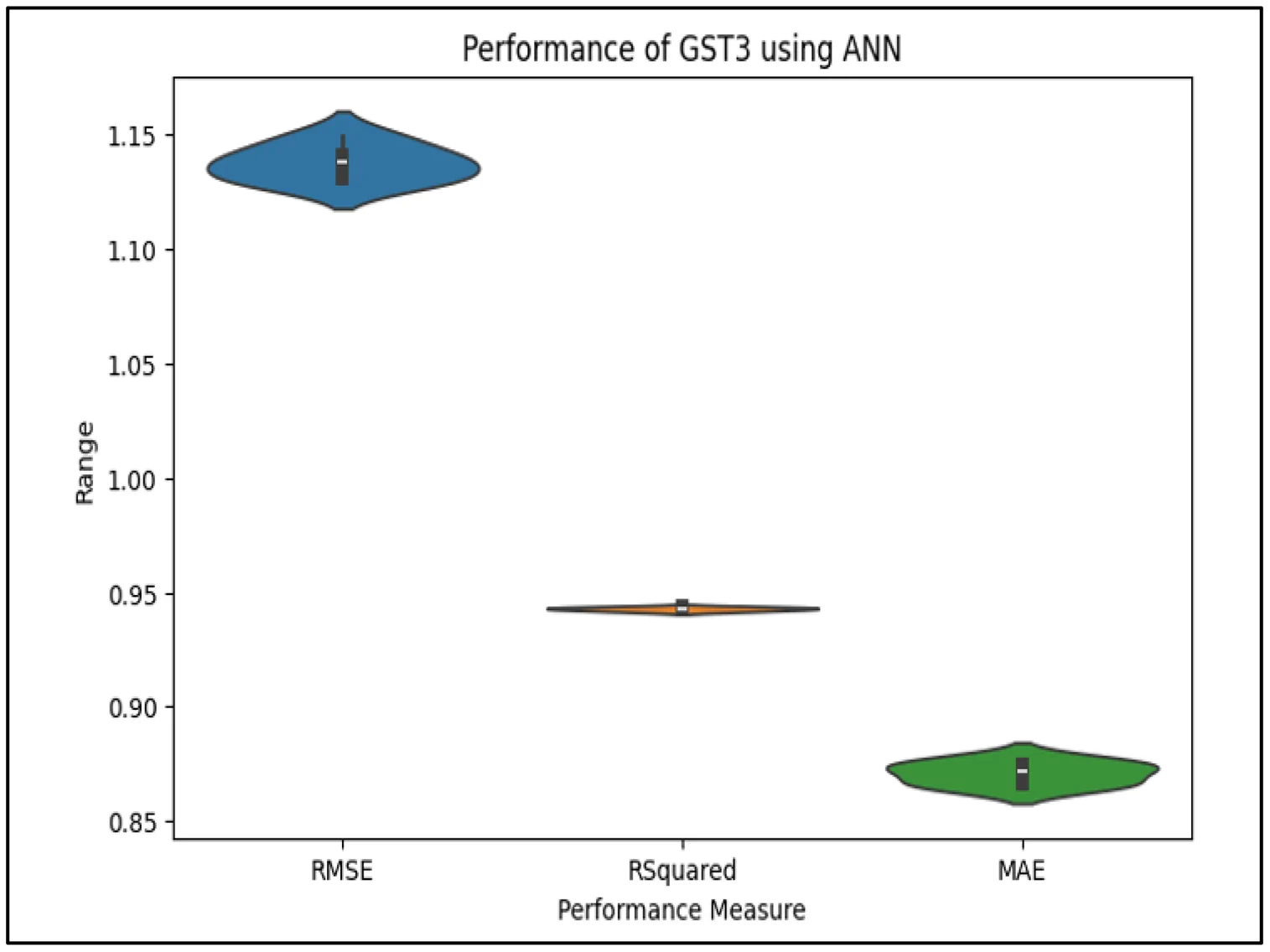
Result of ANN classifier using all parameters.

Overall regression models have performed very well for both types multivariate and univariate. Table 3 indicates both multivariate and univariate results. In this study multivariate regression model performs better than the univariate regression. Both SVM and ANN regression have been implemented for multivariate and univariate. It has been noted that both classifiers performed almost same. From results it was observed that regression models indicated good results for only ambient temperature as a univariate variable as shown in Table 3. Furthermore, less performance was indicated by dewpoint and humidity. It was noted, dew point has indicated squared value less than 0.1 for all three turbines. However, humidity indicated 0.15–0.3 in all three generators. Furthermore Figs 10 and 11 indicate performance of classifiers when performed in a good way.

**Table 3:** Results of Classifiers.

Turbine	AI-bases Regression	Multi-variate Regression			Univariate regression		
		RMSE	MAE	RSquared	RMSE	MAE	RSquared
GST1	SVM	1.079	0.831	0.938	1.223	0.954	0.921
	ANN	1.129	0.817	0.932	1.21	0.942	0.922
GST2	SVM	2.626	0.93	0.698	2.693	1.06	0.682
	ANN	2.67	0.96	0.688	2.689	1.049	0.683
GST3	SVM	1.171	0.892	0.939	1.477	1.19	0.903
	ANN	1.138	0.871	0.943	1.445	1.152	0.908

It was noted that single input variables humidity and dewpoint did not perform well as a univariate regression. However, ambient temperature indicated good results. Almost same results have been found with simple regression process.

## Discussion

One of the most important tasks is to enhance thermodynamic performance of gas turbines. To date hundreds of improvements and modifications have been applied or tested in various ways, to improve the performance of gas turbine. It is expected that following each modification or improvement the finalized product will be able to meet a certain set of requirements by redesigning its components and adding new technology. It has been observed that various gas turbine manufacturers have made improvements with the aim of meeting the customized needs of existing and prospective customers and minimizing design and production costs by leveraging on the knowledge and resources available [20].

**Fig 12:**
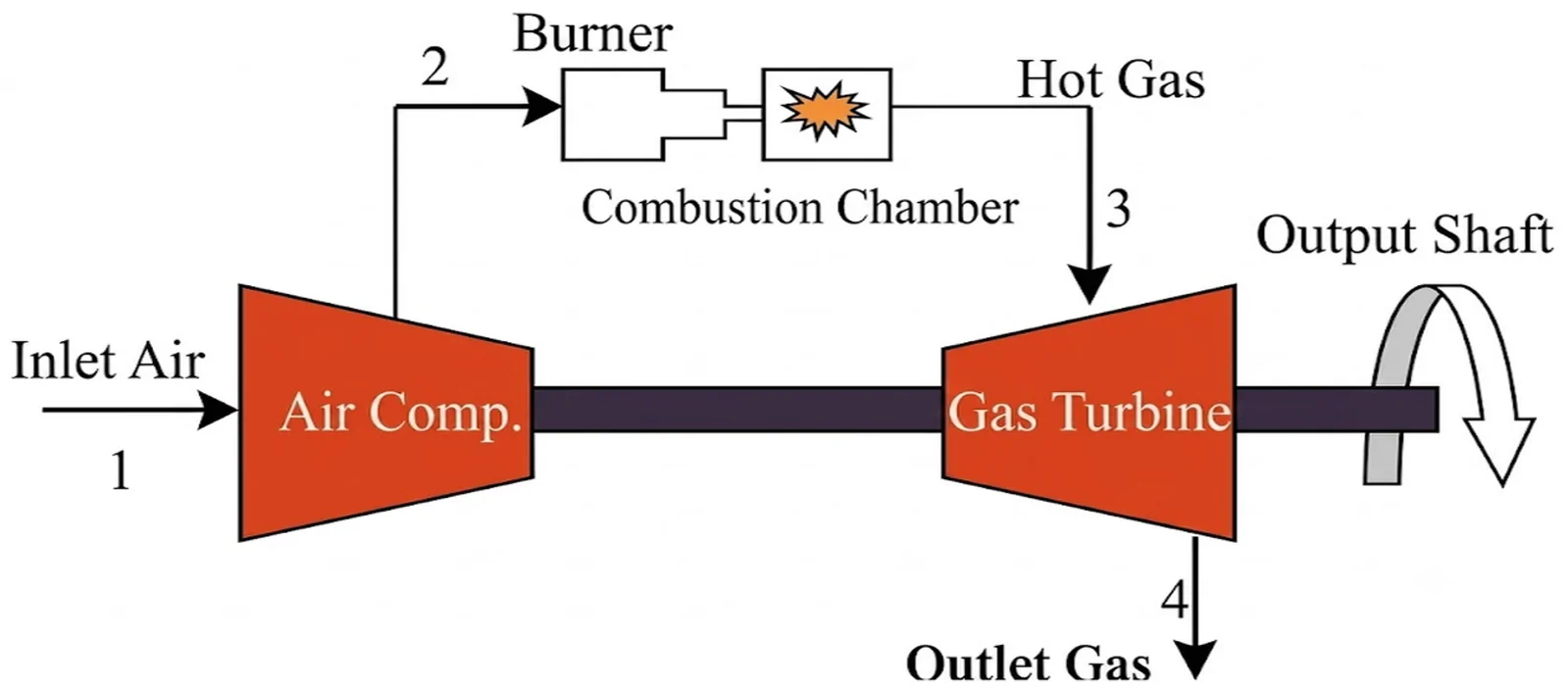
Schematic diagram of gas turbine work cycle [48].

Environmental factors have a great influence on the operation of gas turbine. The compressor will compress the air around it, to a different pressure. Due to compressed air temperature increases. The air that is released from the compressor at a high pressure and temperature is introduced into the combustion chamber at the stage 2 as shown in Fig 12. In fuel-burning device, the fuel and air are mixed together to produce combustion. The combustion chamber can be used for other processes such as mixing, combustion, dilution and cooling even if an unsafe temperature condition occurs at the front of the combustion chamber. Consequently, the temperature of the gas at the turbine inlet (stage 3) as the products of combustion leave the combustion chamber will be mild. A gas turbine is a type of machine that generates mechanical energy on the basis of the heat generated due to the combustion of combustible gases.

The energy conversion process can be divided into two steps: In the first step, the gas expands within the turbine nozzle generating the release of part of the thermal energy it had stored in the form of motion. In second step gas collides with the turning blades and a considerable part of potential energy of the gas is transferred to the blades as kinetic energy. That is, work is performed. The rest of the work that is produced is used in the output flange of the gas turbine while over 50% of the work that is produced is used to drive the compressor. The air volume has to be reduced from stage 4 to stage 1, which is accomplished by cooling the air and releasing the heat to the surrounding. The Brayton cycle is a simple instance of a gas turbine cycle [22].

A variety of applications have been developed for industrial gas turbines, including natural gas pumping/compressor units, peak-loading stations for electrical power, pump sets for firefighting, etc. To generate energy with gas turbines, fuel, compressed air and ignition is needed. Compressed air is an important part of the energy production, based on ambient temperature. One of the main parameters to which the gas turbine performance is directly related to is. Ambient temperature will have a direct effect on the performance of the gas turbine, as the ambient temperature increases, the air density will decrease and so will the performance of the gas turbine.

Now a days, gas turbines are one of the extensively used power generation technology. It was concluded that gas turbine was more appropriate as it could be installed in less time, initial cost per unit output was less, and was environment friendly. In Pakistan, the requirement of electric power is maximum in the summer season. If the ambient temperature is increased, the air density at inlet hood of gas turbines decreases which results in drastic decrease in performance of gas turbines. There is a contrasting ambient condition in the day and night time in Pakistan. In this research article our aim is to determine the relationship between ambient conditions with the performance of gas turbine. Furthermore, our aim is to calculate ambient temperature using which an estimated output of gas turbine can be predicted. Another similar case study has been found. Researchers has found ambient conditions effects on the electricity generation in turkey [20]. In hotter regions, it was found that there is a reduction in electricity generation and vice-versa in cooler regions.

Increase of the ambient temperature leads to a decrease in the density of the incoming air, a decrease in mass flow through the turbine, and thus a decrease in the power output (which is proportional to the mass flow). Gas turbine power is affected by changes in the ambient temperature [23]. As the temperature goes up, the pressure-to-speed ratio of the compressor, at a given RPM, goes down. To examine the influence of the ambient temperature on the generation of power by gas turbine in greater detail. The three gas turbines were chosen for this study. There is also a slight fall in active power with rise in the temperature. This is also evident in the downward sloping trend line. The points are very close to the line, suggesting that there is very little variation in the data and that the relation is strong.

Combustion of gas to produce mechanical energy is done by a gas turbine. The axial flow compressor air input rank is related to the surrounding environment. As per the ISO standard, the efficiency of gas turbine is calculated based on 1.013 (bar) pressure, 60% relative humidity, constant air flow around the turbine and a temperature of 15°C. All these parameters however are known to vary as a function of the installation location, temperature and other environmental conditions. Environmental factors of any particular location are constantly changing in 24 hours of the day, days and months. The climate conditions within Pakistan and especially Nandipur area are different then ISO standard. Therefore, a simple requirement was to find out how climate change was impacting the efficiency of turbine. The results of this study are similar to the research study conducted on the same topic by Karim and Chowdhary [16]. This kind of study is an encouragement to the authors to think about effective and cost-effective ways like cooling, seal and inlet air of turbine. These paths have an impact on the quality of service life and increase the power to fuel ratio [11].

The correlation analysis verified that ambient temperature is the major factor influencing the active power output of all three gas turbine units, which is in line with the thermodynamic theory and the published literature that we have already referred to [47]. Fundamentally, gas turbines are mass flow machines: when ambient temperature increases, air density decreases. So, the compressor inlet mass flow rate also decreases, leading to a dropin pressure ratio, combustion energy input, and turbine output. This is the reason for the strong regression relationships of GT I and GT III. At these turbines, prediction errors were essentially zero, 0. 000329 MW and 0. 00166 MW, respectively. This confirms that ambient temperature, dew point, and humidity are the main factors explaining active power variability at this facility. The weak correlation with dew point and relative humidity agrees with documented results that changes in power production due to humidity are quite small as compared with those brought about by temperature Yet their presence in the multivariate models helped in enhancing overall prediction accuracy, in particular by reflecting the seasonal variations of moisture not covered by temperature alone. Dew point is thermodynamically significant as it denotes the absolute moisture content: if ambient temperature is nearly equal to dew point, compressor inlet condensation occurs, causing slight changes in the effective mass flow and blade aerodynamics which is a strong argument for including it as a predictor variable [15]. Choosing only these three ambient variables instead of operational ones like compressor outlet pressure or fuel composition was done purposely: the objective of this work is to create a prediction model based strictly on weather inputs which can be measured very easily without the need for extra sensors, so that it can be directly used for operational planning at the Nandipur plant. The choice of ambient temperature, dew point, and relative humidity as input variables was made because these parameters change more drastically over a 24-hour period than others like atmospheric pressure and wind speed, which typically have more stable daily patterns. As we are mainly interested in how changes in ambient conditions affect power output, these three parameters reflect the major meteorological factors that can cause variations in the active power output of the gas turbine at the Nandipur plant.

An evaluation of the data collected has shown that the December, January and February months have no data. These three months are considered to be the peak winter months in Pakistan. During winters, the consumption of electricity goes down in Pakistan. Which means, not operating generators on base load. National consumption excluding generator running on base load. In addition, minimum values of data are collected during the month of March, April and November. Maximum number of values has been obtained in June and October which is peak summer season in Pakistan. During the summer season, the electricity consumption is increased to its peak and load shedding (power cut) is also caused during the summer season. The electricity consumption months of May, August and September are less in a period of change of seasons. Furthermore, the ambient temperature at the site selected was also recorded to be between 17 °C and 47 °C during the data collection period. All data taken at “Base Load” (0 dB). Therefore, the designed calculator will be able to predict the performance of gas turbine more precisely, in the range of ambient temperature predicted by the designed calculator from 17°C to 47 °C.

The performance of the ANN and SVM models was relatively comparable, with the SVM displaying a much lower RMSE (1. 079 MW versus 1. 129 MW), lower MAE (0. 831 MW versus 0. 817 MW), and higher coefficient of determination (0. 938 versus 0. 932) for GT I; GT III exhibited virtually identical results for these metrics using the two machine learning algorithms. Again, these coefficient of determination results are consistent with published results such as the coefficient of determination of 0.98 obtained from a seven-variable ANN [47]. It is over twice the number of inputs as used in this study, and a coefficient of determination of 0. 94 obtained via gradient boosting in a recent multi-model comparison of machine learning algorithms [23]. It is encouraging that the present approach with fewer ambient predictor variables yields comparable accuracy. This can be explained by the thermodynamic rationale underpinning the choice of the three predictor variables. The SVM exhibited the superior metrics of a lower RMSE and higher coefficient of determination, which are attributable to the structural risk minimization principle of SVM. Furthermore, it is thought to reduce model overfitting, an issue of concern when modeling operational data with a moderately sized sample. The SVM displayed higher accuracy in the extreme relative prediction errors, while the marginally better mean absolute error indicates it predicts the median errors more effectively than the ANN. GT II displayed much higher prediction errors using the regression and machine learning approaches, and this is caused by unmitigated outliers; better data screening of GT II before modeling should improve results. This study has limitations.

Numerous loop holes for the future are evident in this study. Current study has been done with the data collection at maximum base load. Which indicates this study is suitable only for peak summer season. In future analyses can be performed on different workloads by considering it as a parameter. Simulation studies can be carried out for generators which on different type of generators cover supporting cooling/heating with different materials with can be performed at 60%, 70% and 80% etc. Moreover, simulation and real time studies can be conducted to boost flow and power efficiencies of generators. Data was collected from only one installation and only three ambient input variables were used, and so results may not be generalizable to other climates or turbines. Including more operational variables and larger datasets from multiple sites would be an interesting future direction. Furthermore, Results of this study have not been validated by some other gas turbine due to unavailability of data with same ambient conditions.

## Conclusion

This study demonstrates that accurate prediction of ambient environmental conditions can significantly improve the prediction of gas turbine performance and power output. Reliable estimation of turbine efficiency is essential for effective planning, scheduling, and operational management of power plants. Traditionally, gas turbine performance assessment has relied on ISO standard conditions, which provide output estimations only for specific predefined ambient conditions. However, these standards are limited in their ability to predict real-time operational performance under varying environmental conditions.

To overcome these limitations, this research investigates the relationship between ambient environmental parameters and the performance of gas turbines. The primary objective of the study was to evaluate how factors such as ambient temperature, humidity, pressure, and dew point influence gas turbine output and efficiency. The findings reveal that ambient temperature has a strong negative correlation with gas turbine performance, indicating that higher temperatures substantially reduce turbine output. Humidity exhibited a moderate correlation, whereas ambient pressure and dew point showed comparatively weak relationships with turbine performance.

For this research, real-time operational data were collected over a one-year period from three gas turbines installed at the Nandipur power plant, one of the largest power plants in Pakistan. Although all three turbines were identical in design and specifications, separate regression equations were developed for each turbine to capture their individual operational characteristics. Simple regression analysis techniques were applied to establish predictive relationships between ambient conditions and turbine output.

Based on the derived regression models, a performance prediction calculator was developed. The calculator estimates the performance of GT I, II, and III by using expected ambient temperature and humidity values as input parameters. This tool enables plant operators to forecast turbine output in advance, thereby supporting improved operational planning and energy management.

The developed predictive equations were further validated using actual operational data. The average prediction errors obtained for GT I, GT II, and GT III were 0.000329438, 0.403191003, and 0.00165842, respectively, demonstrating the effectiveness and reliability of the proposed model. The overall results confirm that gas turbine performance is predominantly dependent on ambient temperature. Additionally, Artificial Intelligence (AI)-based techniques were also evaluated and showed promising performance when ambient temperature was used as the primary input variable for prediction.

## Abbreviations

ANN: artificial neural network
SVM: support vector machine
GT: gas turbine
ISO: international organization for standardization
CCPP: combined cycle power plant
RMSE: root mean square error
MAE: mean absolute error
R^2^: coefficient of determination
HDMR: high-dimensional model representation
PSO: particle Swarm optimization
MW: mega watt
GENCOS: genco holding company
UCC: upper Chenab canal
NPGCL: northern power generation company limited
HR: hour
DMM: digital multimeter
AP: active power
RP: reactive power

## References

[1] Basha M, Shaahid SM, Al-Hadhrami L. Impact of Fuels on Performance and Efficiency of Gas Turbine Power Plants. Energy Procedia. 2012;14:558–65. doi: 10.1016/j.egypro.2011.12.975

[2] Jeyaseelan T, Ekambaram P, Subramanian J, Shamim T. A comprehensive review on the current trends, challenges and future prospects for sustainable mobility. Renew Sustain Energy Rev. 2022;157:112073. doi: 10.1016/j.rser.2022.112073

[3] Kolev N, Schaber K, Kolev D. A new type of a gas–steam turbine cycle with increased efficiency. Appl Therm Eng. 2001;21(4):391–405.

[4] Vellaiyan S. Optimization of hydrogen-enriched biodiesel-diesel dual-fuel combustion with EGR for sustainable engine performance. Int J Hydrogen Energy. 2025;128:85–94. doi: 10.1016/j.ijhydene.2025.04.239

[5] Salilew WM, Karim ZAA, Lemma TA. Investigation of fault detection and isolation accuracy of different machine learning techniques with different data processing methods for gas turbine. Alex Eng J. 2022;61(12):12635–51.

[6] Ragland TL. Industrial Gas Turbine Performance Uprates: Tips, Tricks, and Traps. J Eng Gas Turbines Power. 1998;120(4):727–34. doi: 10.1115/1.2818460

[7] Kim B, Mai TD, Vo D-T, Lee HK, Jeong Y, Chang S, et al. Hydrogen combustion in two-stage high-pressure gas turbine—thermal and flow simulations for performance comparison with fossil fuels. Int J Engine Res. 2024;25(3):513–26. doi: 10.1177/14680874231196987

[8] Igumenov I, Aksenov A. Thermal barrier coatings on gas turbine blades: Chemical vapor deposition. Therm Eng. 2017;64(12):865–73.

[9] Du Z, You R, Li H, Tian C, Quan S. Geometric optimization for low-degradation film-cooling holes at the leading edge of turbine blades. Appl Therm Eng. 2025;278:127222.

[10] Ahsan S, Lemma TA, Hashmi MB, Liang X. Investigation of operational settings, environmental conditions, and faults on the gas turbine performance. Meas Sci Tech. 2024;35(12):125902.

[11] Goucem M. Influence of the Ambient Temperature on the Efficiency of Gas Turbines. FDMP. 2024;20(10):2265–79. doi: 10.32604/fdmp.2024.052365

[12] Liu Z, Karimi IA. Gas turbine performance prediction via machine learning. Energy. 2020;192:116627. doi: 10.1016/j.energy.2019.116627

[13] Pinilla Fernandez DA, Foliaco B, Padilla RV, Bula A, Gonzalez-Quiroga A. High ambient temperature effects on the performance of a gas turbine-based cogeneration system with supplementary fire in a tropical climate. Case Stud Therm Eng. 2021;26:101206. doi: 10.1016/j.csite.2021.101206

[14] Sammour AA, Komarov OV, Lattieff FA, Qasim MA, Saleh AY. Influence of Surrounding Air Temperature and Humidity upon the Performance of a Gas Turbine Power Plant. ARFMTS. 2024;112(1):22–37. doi: 10.37934/arfmts.112.1.2237

[15] Sohani A, Farasati Y, Sayyaadi H. A systematic approach to find the best road map for enhancement of a power plant with dew point inlet air pre-cooling of the air compressor. Energy Convers Manag. 2017;150:463–84. doi: 10.1016/j.enconman.2017.08.028

[16] Kumar S, Singh O. Effect of gas/steam turbine inlet temperatures on combined cycle having air transpiration cooled gas turbine. Inst Eng India Ser C. 2012;93(4):297–305.

[17] Dellenback PA. Improved Gas Turbine Efficiency Through Alternative Regenerator Configuration. J Eng Gas Turbines Power. 2002;124(3):441–6. doi: 10.1115/1.1451843

[18] Ünver Ü, Keleşoğlu A. A novel method for prediction of gas turbine power production degree-day method. Therm Sci. 2018;22:S809–17.

[19] Tüfekci P. Prediction of full load electrical power output of a base load operated combined cycle power plant using machine learning methods. Int J Electr Power Energy Syst. 2014;60:126–40. doi: 10.1016/j.ijepes.2014.02.027

[20] Ashraf WM, Keshavarzzadeh AH, Alshehri AS, Jumah A bin, Debnath R, Dua V. Domain-informed operation excellence of gas turbine system with machine learning. Energy Convers Manag. 2026;348:120789. doi: 10.1016/j.enconman.2025.120789

[21] Manatura K, Rummith N, Chalermsinsuwan B, Samsalee N, Chen WH, Phookronghin K, et al. Gas turbine heat rate prediction in combined cycle power plant using artificial neural network. Therm Sci Eng Prog. 2025;59:103301.

[22] Abdelsattar M, A Ismeil M, Menoufi K, AbdelMoety A, Emad-Eldeen A. Evaluating Machine Learning and Deep Learning models for predicting Wind Turbine power output from environmental factors. PLoS One. 2025;20(1):e0317619. doi: 10.1371/journal.pone.0317619 39847588 PMC11756752

[23] Lakhdar L, Naas TT. The machine learning for predicting gas turbine performance in naval vessels. J Eng Technol Ind Appl. 2024;10(49):187–93.

[24] Surase RS, Konijeti R, Chopade RP. Thermal performance analysis of gas turbine power plant using soft computing techniques: a review. Eng Appl Comput Fluid Mech. 2024;18(1):2374317. doi: 10.1080/19942060.2024.2374317

[25] Lim JT, Habibullah A, Ng EYK. Towards a Digital Twin for Gas Turbines: Thermodynamic Modeling, Critical Parameter Estimation, and Performance Optimization Using PINN and PSO. Energies. 2025;18(14):3721. doi: 10.3390/en18143721

[26] Huang Q, Gu X, Shao J, Yang S. A digital-twin assisted performance prediction model for industrial gas turbines. In: International conference on the Efficiency and Performance Engineering Network. Springer; 2023.

[27] Ma Y, Zhu X, Lu J, Yang P, Sun J. Construction of Data-Driven Performance Digital Twin for a Real-World Gas Turbine Anomaly Detection Considering Uncertainty. Sensors (Basel). 2023;23(15):6660. doi: 10.3390/s23156660 37571444 PMC10422313

[28] Hu M, He Y, Lin X, Lu Z, Jiang Z, Ma B. Digital twin model of gas turbine and its application in warning of performance fault. Chin J Aeronaut. 2023;36(3):449–70. doi: 10.1016/j.cja.2022.07.021

[29] Ramzan M, Shafique A, Abbas S, Ali R, Lamoudan T, Jan R, et al. Mathematical analysis of Prandtl number with Artificial neural network and fractional operator for nanofluid flow. Case Stud Therm Eng. 2025;71:106164. doi: 10.1016/j.csite.2025.106164

[30] Huang H, Zhang W, Xu Y, Zhang H, Zou J. A method for PWM frequency harmonic suppression using zero-vector switching SVPWM strategy in three-level inverter drive systems. IEEE J Emerg Sel Top Power Electron. 2025;13(2):1482–91.

[31] Li X, Wang Y, Ma X, Chen L, Francisco MDV, Zhang X. PIC-MCC simulation of single pulse DBD discharge evolution process. Plasma Sources Sci Technol. 2026;35(1):015021. doi: 10.1088/1361-6595/ae33ed

[32] Yang H, Wang F, Zhang H, Guo L, Hu L, Wang L, et al. Solution synthesis of layered van der Waals (vdW) ferromagnetic CrGeTe3 nanosheets from a non-vdW Cr2Te3 template. J Am Chem Soc. 2020;142(9):4438–44. 10.1021/jacs.9b13492 31976663

[33] Ren F, Zhuang P-Z, Chen X, Yu H-S, Yang H. Physics-Informed Extreme Learning Machine (PIELM) for Stefan problems. Comput Methods Appl Mech Eng. 2025;441:118015. doi: 10.1016/j.cma.2025.118015

[34] Xiao D, Chen W, Tang H, Xiao H, Zuo Y, Chu Z, et al. Economic limits of horizontal spacing for U-shaped closed-loop geothermal well conversion from two abandoned petroleum wells. Renew Energy. 2026;263:125546. doi: 10.1016/j.renene.2026.125546

[35] Wang Q, Xiao D, Tang G, Lu Z, Ge X, Zou C, et al. A latent-heat-coupled wellbore heat-transfer model with phase-change materials. Phys Fluids. 2026;38(4). doi: 10.1063/5.0324428

[36] Nazar M, Abbas S, Asghar S, Saleem S, Abutuqayqah H, AL Garalleh H, et al. Comparison of unsteady MHD flow of second grade fluid by two fractional derivatives. Numeri Heat Transf Part A: Appl. 2026;87(1):2374536. doi: 10.1080/10407782.2024.2374536

[37] Rafique U, Nazar M, Abbas S, Elnaggar GR, Arishi A, Abdullaeva B. Investigation of a novel thermal and mass stratification effect on second grade fluid: Applications in industrial heat transfer systems. Case Stud Therm Eng. 2025;73:106399. doi: 10.1016/j.csite.2025.106399

[38] Abbas S, Gilani SFF, Nazar M, Fatima M, Ahmad M, Nisa ZU. Bio-convection flow of fractionalized second grade fluid through a vertical channel with Fourier’s and Fick’s laws. Mod Phys Lett B. 2023;37(23):2350069. doi: 10.1142/s0217984923500690

[39] Abbas S, Nazar M, Gillani SFF, Naveed M, Ahmad M, Nisa ZU. A CPC fractional model of the heat and mass transport mechanism in carbon nanotubes with slip effects on velocity. Mod Phys Lett B. 2024;38(13):2450100.

[40] Abbas S, Inam I, Alharthi AM, AL-Khasawneh MAS, Az-Zo’bi EA, Garalleh HA. Fractional magnetohydrodynamic Casson fluid flow with thermal radiation and buoyancy effects: a constant proportional Caputo model. Bound Value Probl. 2025;2025(1). doi: 10.1186/s13661-025-02036-4

[41] Abbas S, Nisa ZU, Nazar M, Metwally ASM, Kędzia K, Jan AZ, et al. Effect of chemical reaction on MHD Casson natural convection flow over an oscillating plate in porous media using Caputo fractional derivative. Int J Therm Sci. 2025;207:109355.

[42] Abbas S, Ramzan M, Inam I, Saleem S, Nazar M, Abduvalieva D, et al. Analysis of fractionalized Brinkman flow in the presence of diffusion effect. Sci Rep. 2024;14(1):22507. doi: 10.1038/s41598-024-72785-2 39341809 PMC11439061

[43] Nabi FG, Ghaffar A, Ishfaq K, Sundaraj K, Yang G. Statistical and machine learning analysis of respiratory sounds for multilevel asthma severity classification. J Eng Res. 2026. doi: 10.1016/j.jer.2026.04.021

[44] Nabi FG, Sundaraj K, Lam CK. Identification of asthma severity levels through wheeze sound characterization and classification using integrated power features. Biomed Signal Process Control. 2019;52:302–11. doi: 10.1016/j.bspc.2019.04.018

[45] Nabi FG, Sundaraj K, Iqbal MS, Shafiq M, Palaniappan R. A telemedicine software application for asthma severity levels identification using wheeze sounds classification. Biocybern Biomed Eng. 2022;42(4):1236–47.

[46] Lin Q, Li G, Pan X, Lin Y, Nabi FG, Li S, et al. SDNAL-Seg: multi-scale selective downsampling and non-adjacent layers guidance for medical image segmentation. SIViP. 2026;20(7). doi: 10.1007/s11760-026-05376-5

[47] De Sa A, Al Zubaidy S. Gas turbine performance at varying ambient temperature. Appl Therm Eng. 2011;31(14–15):2735–9.

[48] Kumar S, Singh O. Effect of Gas/Steam Turbine Inlet Temperatures on Combined Cycle Having Air Transpiration Cooled Gas Turbine. J Inst Eng India Ser C. 2012;93(4):297–305. doi: 10.1007/s40032-012-0046-9

